# Supplementary material for: Structure and mechanism of DNA delivery of a gene transfer agent
Source: Nat Commun. 2020 Jun 15;11:3034. doi: 10.1038/s41467-020-16669-9 (PMC7296036; doi:10.1038/s41467-020-16669-9)
Supplement: Supplementary file 1 — Supplementary Information [file 41467_2020_16669_MOESM1_ESM.pdf]

# Supplementary material for:

# Structure and mechanism of DNA delivery of gene transfer agent

Pavol Bárdy[1], Tibor Füzik[2], Dominik Hrebik[2], Roman Pantůček[1], J. Thomas Beatty[3], Pavel Plevka[2]*

Author Affiliations:

1 – Department of Experimental Biology, Faculty of Science, Masaryk University, Kamenice 5, 625 00, Brno, Czech Republic

2 – Central European Institute of Technology, Masaryk University, Kamenice 5, 625 00, Brno, Czech Republic

3 – Department of Microbiology and Immunology, University of British Columbia, 2350 Health Sciences Mall, Vancouver, V6T 1Z3, Canada.

* Corresponding author: pavel.plevka@ceitec.muni.cz

**Supplementary material includes:**

- **Supplementary Figures**
- **Supplementary Tables**
- **Supplementary References**

## Supplementary Figures

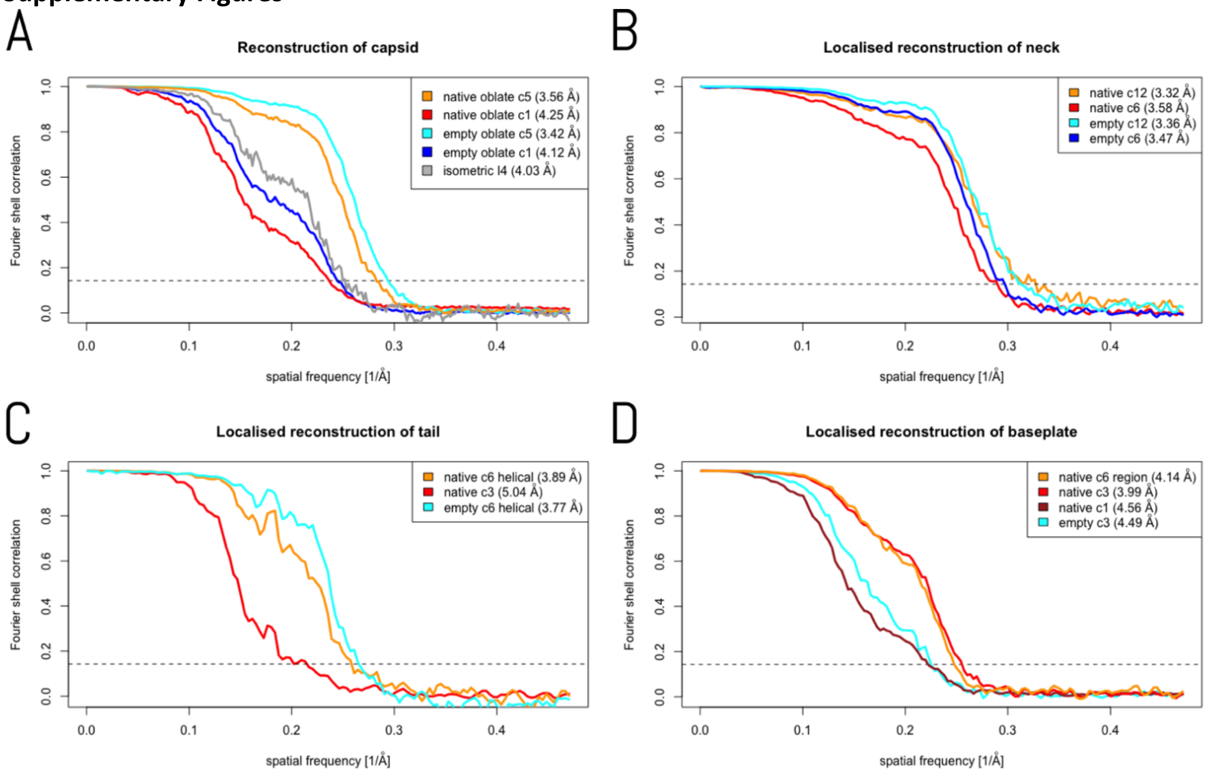


**Supplementary Figure 1. FSC curves of cryo-EM reconstructions of RcGTA in various states.**

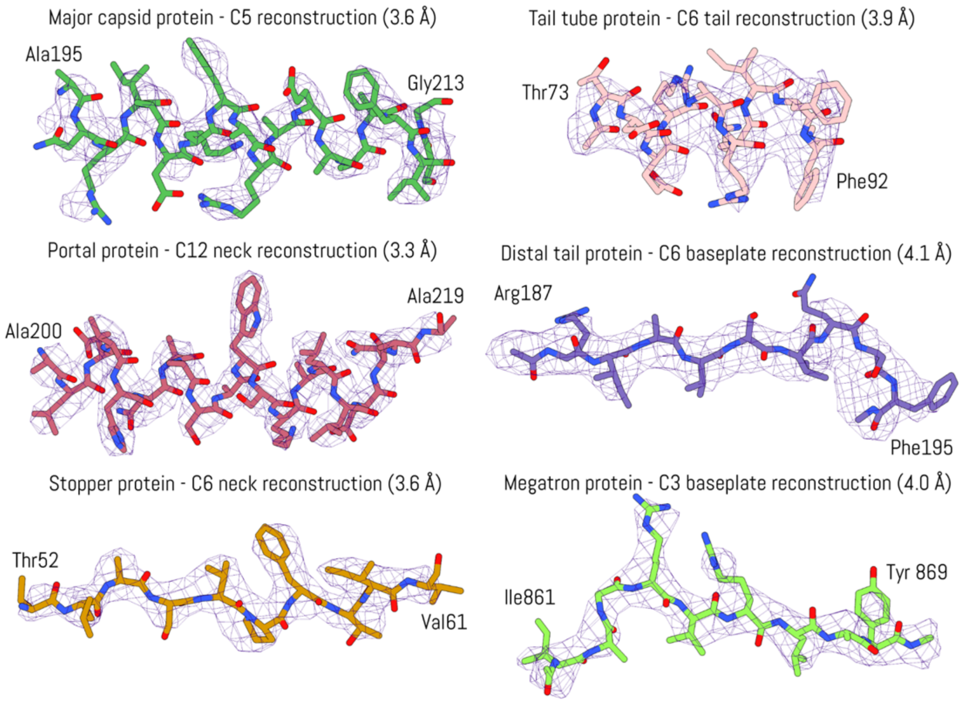


**Supplementary Figure 2. Examples of fit of PDB structures of RcGTA into cryo-EM reconstructions.**
Segments of protein structures and their fit into the corresponding cryo-EM densities.

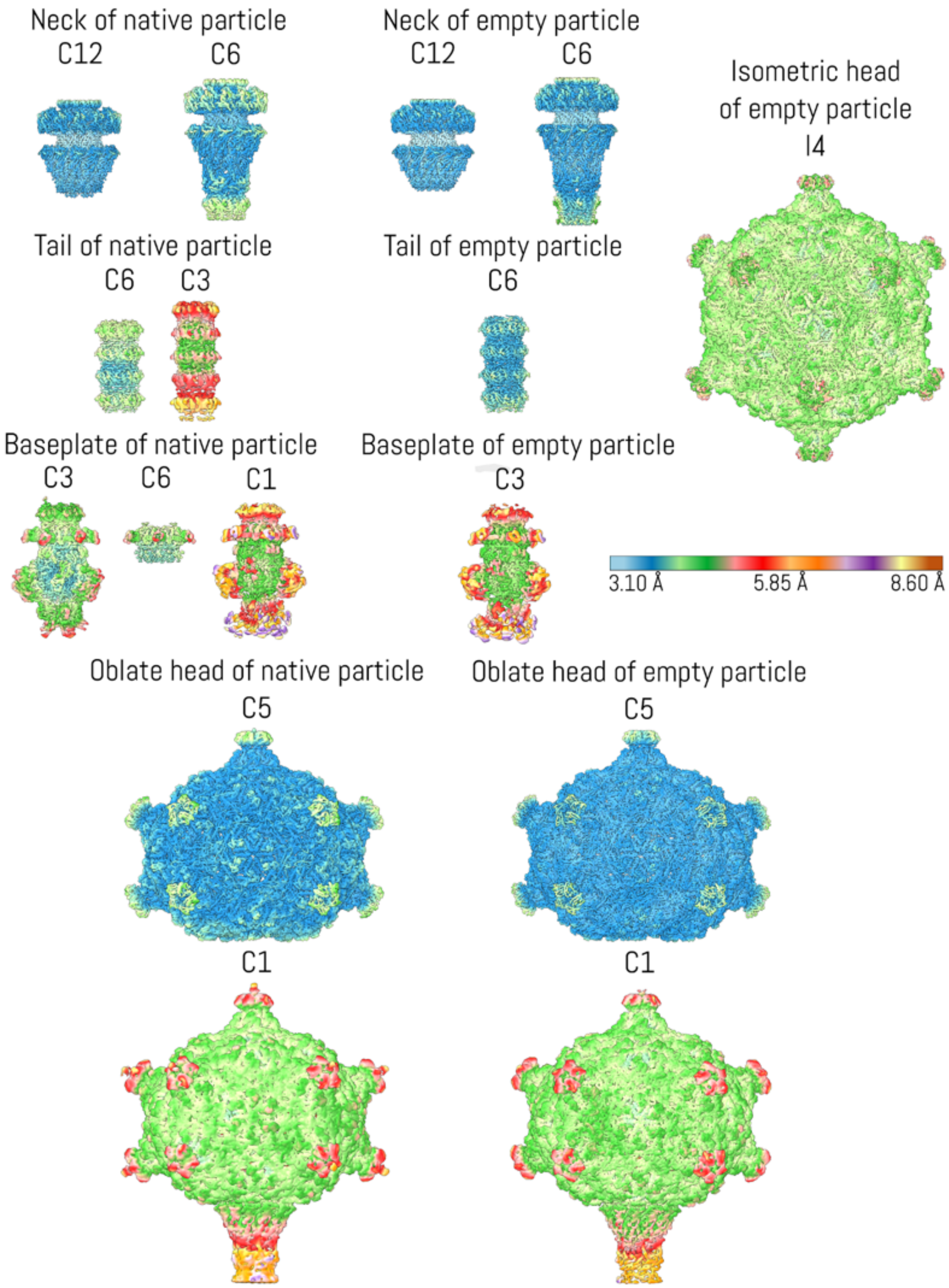


**Supplementary Figure 3. Local resolution maps of RcGTA structures.** Cryo-EM reconstructions of native and empty RcGTA particles and their parts are colored according to local resolution as determined using the program RELION(*1*). The color-coding scheme is based on a twelve-component palette prepared using the program ChimeraX(*2*).

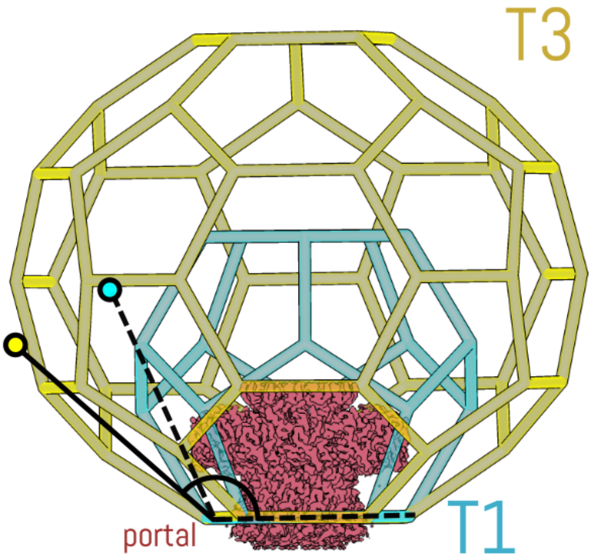


**Supplementary Figure 4. Geometries of RcGTA capsid proteins and portal complex prevent formation of T = 1 head.** Mesh corresponding in size to icosahedral T = 1 head built from RcGTA capsid proteins is shown in blue and that corresponding to the oblate head based on T = 3 quasi-icosahedral symmetry is shown in yellow. The portal complex, shown in space-filling representation in red, fits into the icosahedral T = 1 capsid, but would require different parts of capsid proteins to bind to the portal complex than those in capsids with higher T numbers.

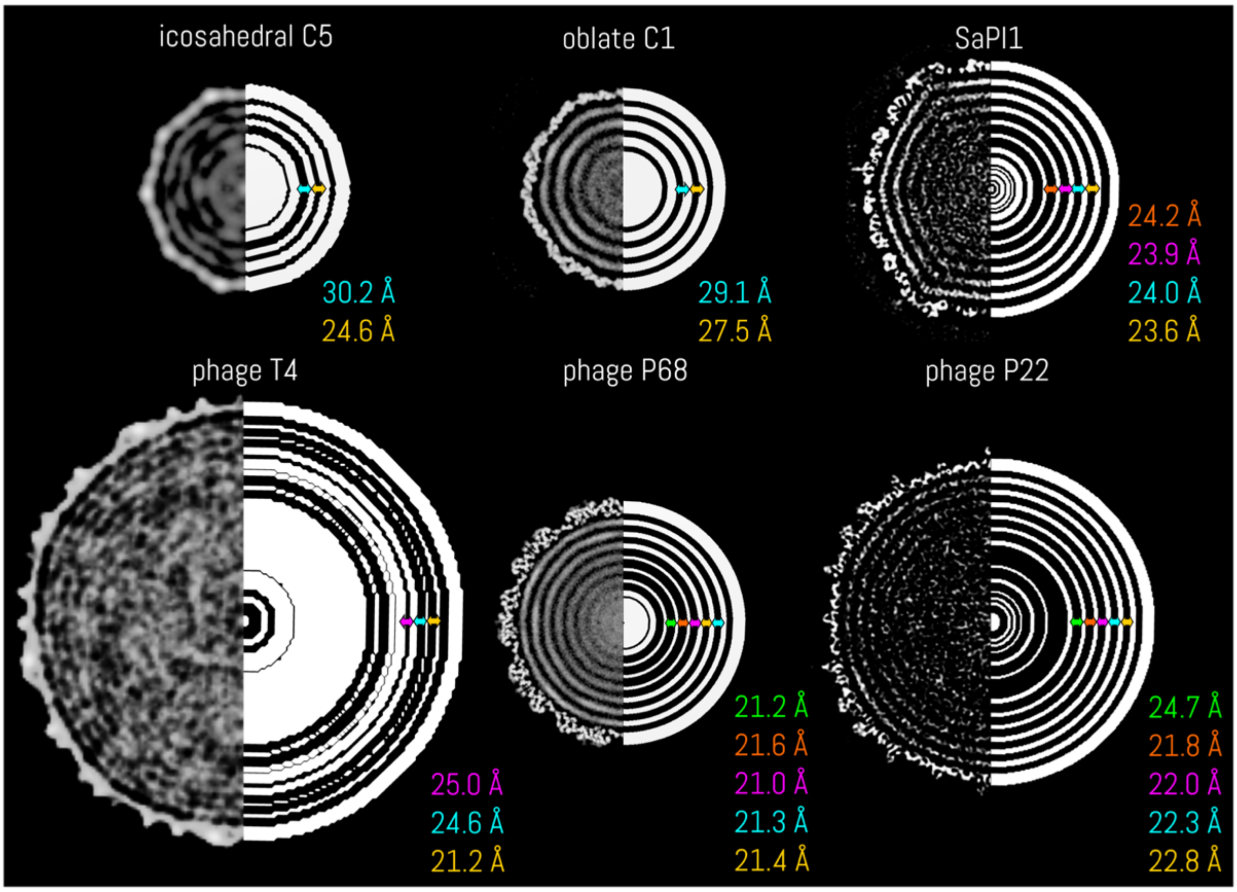


**Supplementary Figure 5. Density of DNA in RcGTA head is lower than those of tailed phages.** The spacing of DNA layers in icosahedral (A) and oblate (B) heads of RcGTA, and the head of phage 80α transducing staphylococcal pathogenicity island I (C), phage T4 (D), phage P68 (E), and phage P22 (F)(*3-6*). The left halves of the individual panels show central slices of cryo-EM maps of the heads, and the right halves show radial averages of the corresponding reconstructions. Distances between the centers of masses of consecutive layers of density were measured.

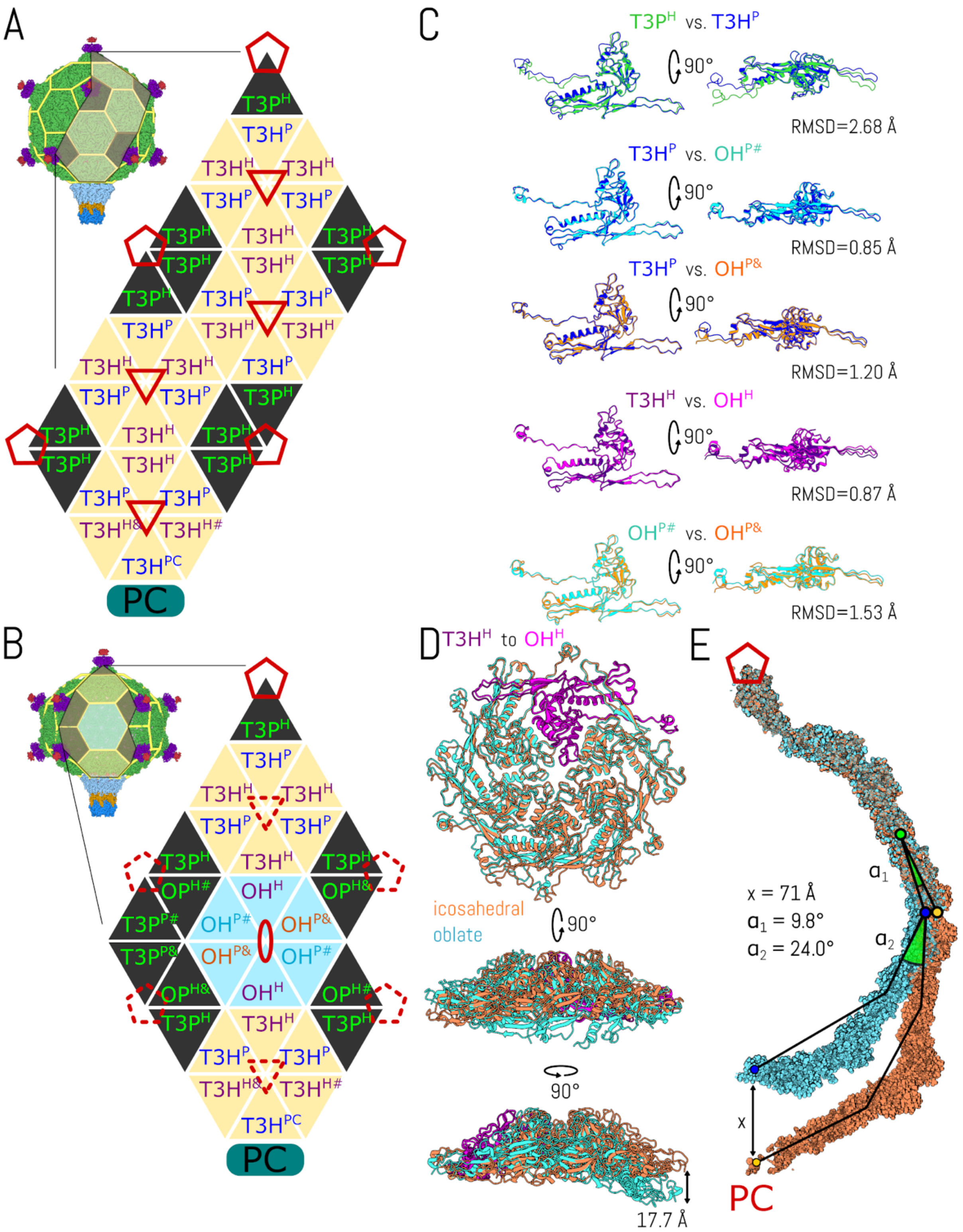


**Supplementary Figure 6: Differences in conformations of capsid proteins enable the formation of T = 3 quasi-icosahedral and oblate heads of RcGTA.** Schemes of distributions of capsid proteins with different conformations in icosahedral (A) and oblate (B) heads of RcGTA. The diagrams show one fifth of each of the capsids with portal complexes positioned at the bottom and indicated by blue ovals labelled PC. The head with T = 3 quasi-icosahedral symmetry (A) contains major capsid proteins in three general conformations: subunits forming pentamers interacting with hexamers ($T3P^H$), hexamers interacting with pentamers ($T3H^P$), and hexamers interacting with hexamers ($T3H^H$). The

presence of the portal complex affects the structures of three neighboring capsid proteins ($T3H^{PC}$, $T3H^{H\#}$, and $T3H^{H\&}$). (B) The oblate head contains subunits with additional conformations, including a quasi-hexamer of the capsid proteins positioned on a twofold axis (light blue). It is built from capsid proteins in three conformations: subunits interacting with neighboring hexamers ($OH^{H}$), and two distinct conformations of subunits interacting with pentamers ($OH^{P\#}$ and $OH^{P\&}$). In addition, the oblate particle includes two conformations of subunits that mediate inter-pentamer interactions ($OP^{P\#}$ and $OP^{P\&}$). Pentagons and triangles indicate positions of fivefold and threefold axes of icosahedral symmetry. The positions of local quasi-symmetry axes in the oblate capsid are indicated by dashed pentagons and triangles, respectively. The position of the twofold symmetry axis of the oblate head is indicated by a red oval. (C) Comparisons of conformations of capsid proteins that form T = 3 quasi-icosahedral and oblate capsids of RcGTA. The notation of subunits is the same as that used in panel (A). Superimposed subunits are shown in two orientations rotated by 90° around the X-axis relative to each other. RMSD values of the corresponding atoms of the superimposed structures are indicated. (D) Comparison of structures of quasi-hexamers positioned on threefold axis of T = 3 quasi-icosahedral capsid (orange) and that positioned on twofold axis of oblate head (cyan). Subunits $T3H^{H}$ (purple) and $OH^{H}$ (magenta) from the two quasi-hexamers were superimposed. The superimposed quasi-hexamers are shown in top and side views to demonstrate that the icosahedral quasi-hexamer is planar and the one positioned on the twofold axis of the oblate head is bent. (E) Comparison of curvature of oblate and icosahedral capsids of RcGTA. Central slices of one fifth of icosahedral (orange) and oblate (cyan) capsids are shown. The two structures were superimposed based on their $T3P^{H}$ subunits located opposite to the portal complex.

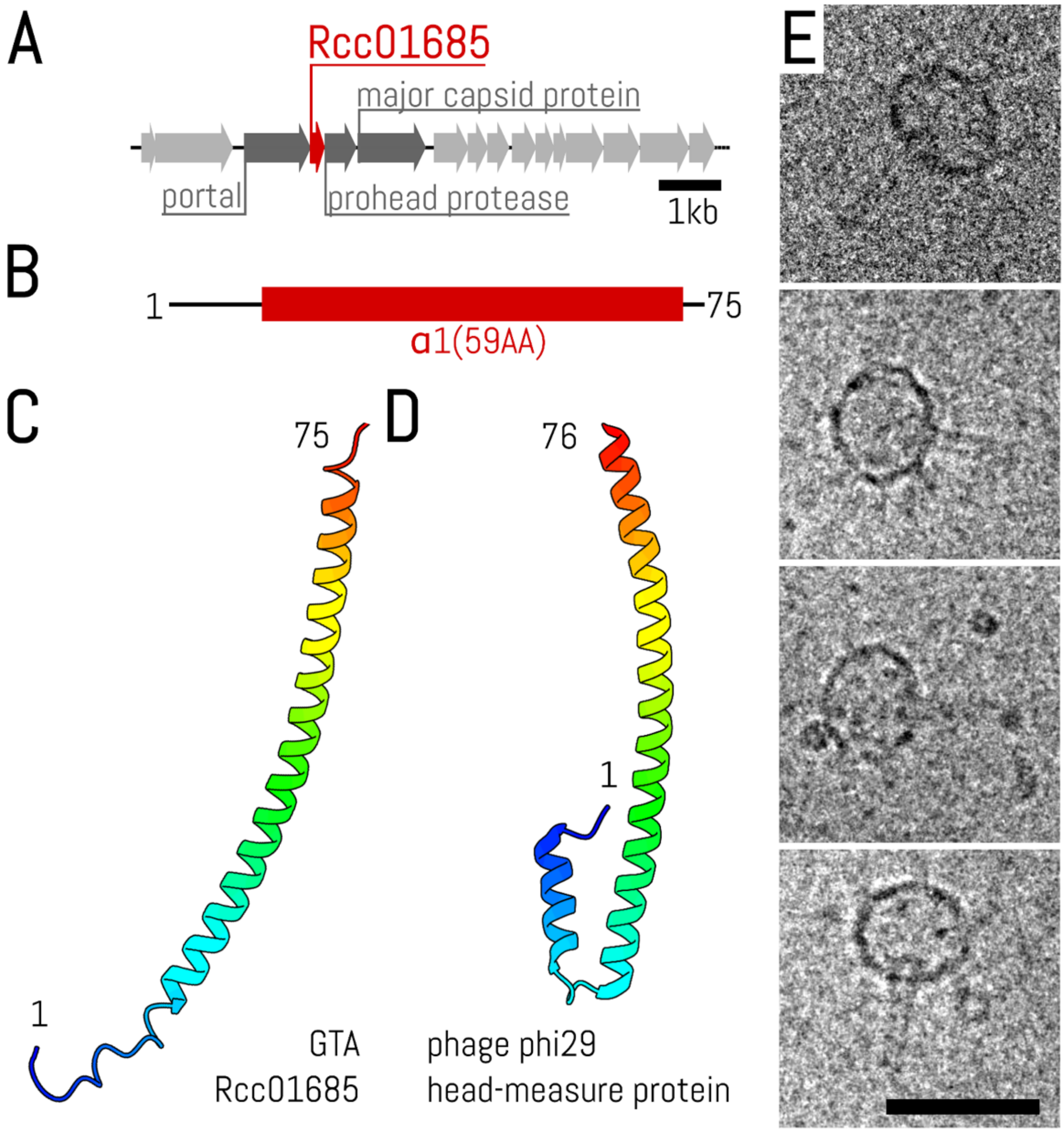


**Supplementary Figure 7. Rcc01685 is not a scaffolding protein that determines the oblate shape of the RcGTA head.** (A) Rcc01685 is encoded within the head assembly module of RcGTA. (B) Secondary structure prediction indicates that Rcc01685 forms a long α-helix. (C) Cartoon representation of model of Rcc01685 structure colored from N-terminus in blue to C-terminus in red. The model was generated using the program RaptorX(*7*). (D) Structure of head-measure protein of phage phi29 (PDB 1no4). (E) *R. capsulatus* strain SB1003 with deleted Rcc01685 produced particles with oblate heads. All the observed particles lacked DNA. In total, eleven particles were observed. Scale bar represents 50 nm.

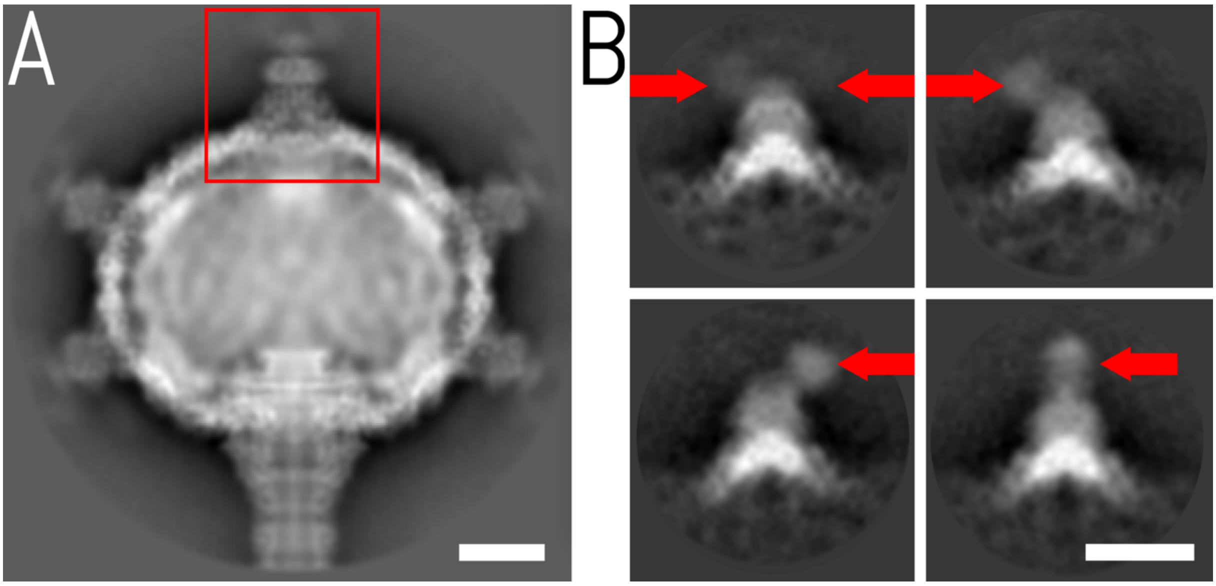


**Supplementary Figure 8. Head spikes of RcGTA are flexible.** (A) Reference-free two-dimensional class average of oblate RcGTA head showing diffuse densities corresponding to head fibers, indicating that they adopt multiple conformations. The reconstruction is based on 35,966 particle images. (B) Reference-free two-dimensional class averages of individual head spikes pointing in various directions. Head fibers adopt several different conformations as indicated by arrows. The reconstructions, from top left to bottom right, are based on 1,126; 962; 502; and 545 particle images, respectively. Scale bars correspond to 10 nm.

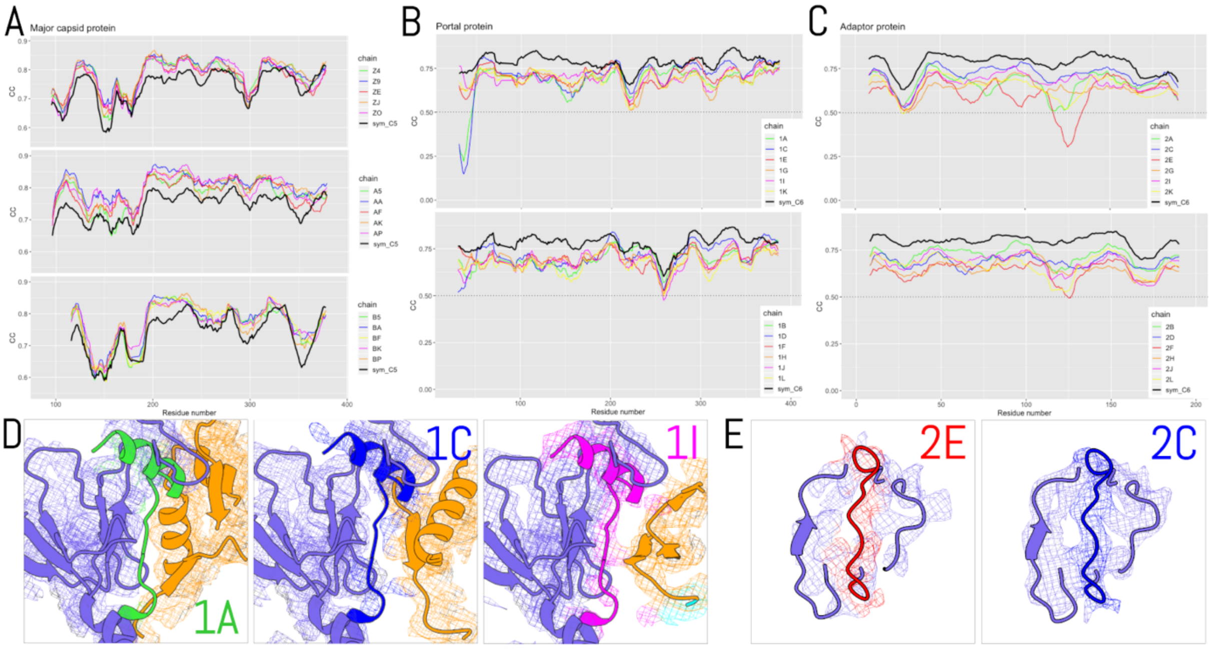


**Supplementary Figure 9. Asymmetric structure of RcGTA tail-capsid interface.** (A-C) Plots of correlation coefficients comparing values from asymmetric cryo-EM map of RcGTA with electron densities calculated from structures of capsid (A), portal (B), and adaptor (C) proteins built into symmetrized maps. The plots were smoothened by a 15-residue sliding window average. Lines in the plots are differentiated by color and labeled by chain according to the RcGTA structure (PDB 6TBA). Low values in the diagrams indicate that the asymmetric structures deviate from the symmetrized one. The black line represents the cross-correlation of the symmetrized structure with symmetrized cryo-EM map. For major capsid proteins, the plots are shown separately for each of the three capsid protein orientations that interact with the neck region. For the portal and adaptor, data from two unique chains modelled to the C6 symmetrized map are shown separately in the upper and bottom plots. (D) Cartoon representation of regions of portal proteins fitted into asymmetric map with correlation coefficient lower than 0.5 (chains 1A and 1C) and subunit with the highest correlation coefficient (1I). Capsid proteins are shown in orange, portal proteins in light blue and the region of the portal protein in an alternative conformation in green (chain 1A) and blue (chain 1C). Cryo-EM maps are shown as mesh colored according to the chain it belongs to. (E) Cartoon representation of part of the adaptor protein that fits into the asymmetric map with correlation coefficient lower than 0.5 (chain 2E) and subunit with the highest correlation coefficient (chain 2C). The region of adaptor protein 2E with a poor fit into the asymmetric density is shown in red.

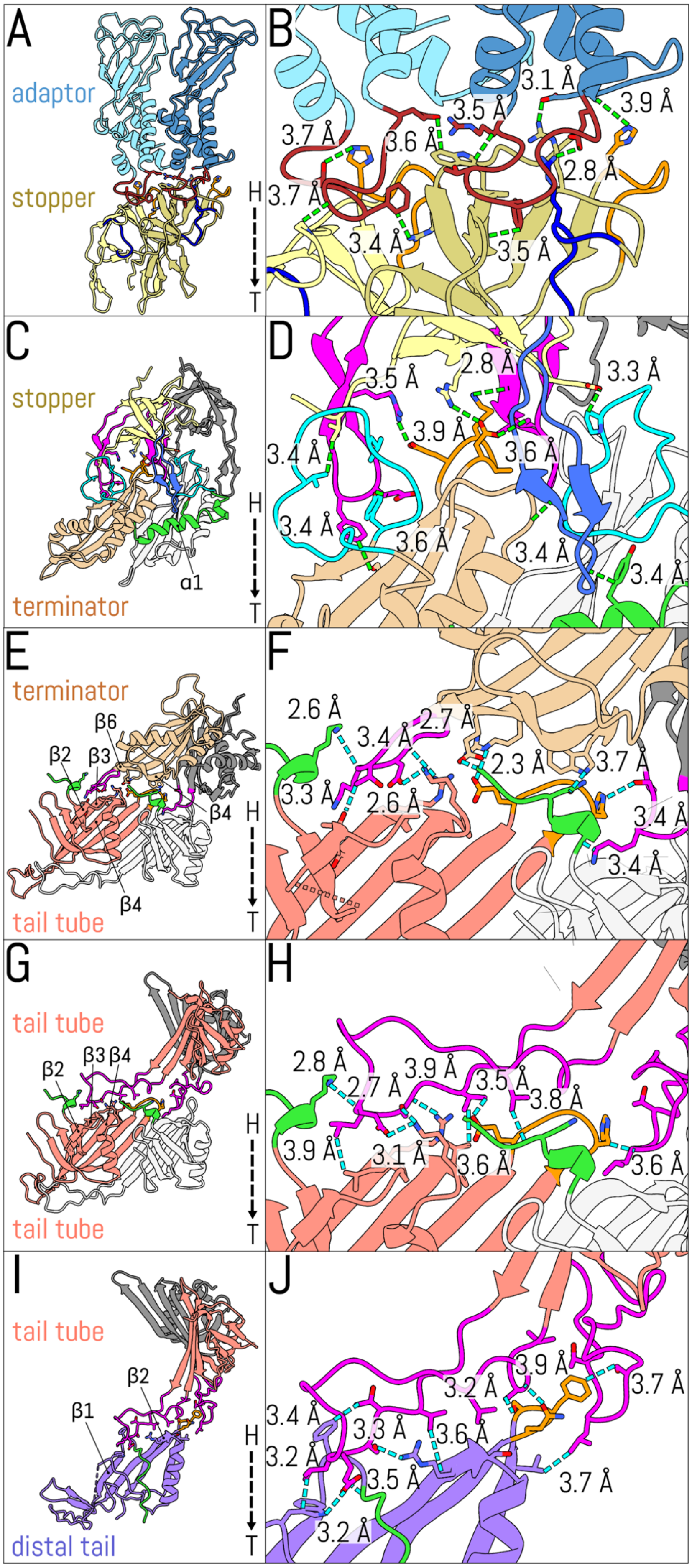
A
adaptor
stopper
H
T
B
3.1 Å
3.9 Å
3.5 Å
3.7 Å
3.6 Å
2.8 Å
3.7 Å
3.4 Å
3.5 Å
C
stopper
α1
terminator
D
2.8 Å
3.3 Å
3.5 Å
3.9 Å
3.6 Å
3.4 Å
3.4 Å
3.6 Å
3.4 Å
3.4 Å
E
terminator
β6
β2
β3
β4
β4
tail tube
F
2.6 Å
3.4 Å
2.7 Å
2.3 Å
3.7 Å
3.3 Å
2.6 Å
3.4 Å
3.4 Å
G
tail tube
β2
β3
β4
tail tube
H
2.8 Å
2.7 Å
3.9 Å
3.5 Å
3.8 Å
3.9 Å
3.1 Å
3.6 Å
3.6 Å
I
tail tube
β2
β1
distal tail
J
3.9 Å
3.2 Å
3.7 Å
3.4 Å
3.3 Å
3.6 Å
3.2 Å
3.5 Å
3.7 Å
3.2 Å

**Supplementary Figure 10. Details of interfaces between tail proteins of RcGTA.**

**(AB) Interface between adaptor and stopper proteins.** (A) Reduction in symmetry from twelvefold of adaptor complex to sixfold of stopper proteins is mediated by adaptor loops of adaptor proteins which have different conformations in odd and even subunits. Adaptor proteins are shown in light and steel blue and stopper proteins in yellow and khaki. Adaptor loops of adaptor proteins are highlighted in red. The core helix and short loop of the stopper protein are shown in dark blue and orange, respectively. The head-to-tail direction is indicated with an arrow. (B) Detail of interface between adaptor and stopper proteins. Side chains of interacting residues are shown in stick representation and selected distances are indicated.

**(CD) Interface between stopper and tail terminator proteins.** (C) Interaction of the stopper proteins (yellow and grey) and tail terminator proteins (beige and white). The long loop of the yellow stopper protein is shown in magenta and the insertion loop in blue. The stopper protein, displayed in grey, contains the long loop highlighted in magenta. The terminator protein, displayed in beige, has the insertion loop highlighted in cyan and short loop in orange. The terminator protein, displayed in white, contains the N-terminal helix highlighted in green and insertion loop in cyan. (D) Detail of stopper-tail terminator protein interface. Side chains of interacting residues are shown in stick representation and selected interatomic distances are shown.

**(EF) Interface between tail terminator and tail tube proteins.** (E) Interaction of tail terminator proteins (beige and grey) with tail tube protein (salmon and white). Long loops of tail terminator proteins are highlighted in magenta. The N-terminus of the salmon-colored tail tube protein is shown in green, and the short loop in orange. The tail tube protein, which is shown in white, has the N-terminus highlighted in green. (F) Detail of interface between the terminator and tail tube proteins. Distances between interacting residues are indicated.

**(GH) Interface between consecutive discs of tail tube proteins.** (G) Interactions of tail tube proteins from two consecutive discs in the RcGTA tail. Long loops of tail tube proteins proximal to the head are shown in magenta. The N-terminus of the salmon-colored tail tube protein distal from the head is shown in green, and the short loop in orange. The tail tube protein, which is shown in white, has its N-terminus highlighted in green. (H) Detail of interface between two tail tube proteins. Distances of selected interacting residues are indicated.

**(IJ) Interface between tail tube and distal tail proteins.** (A) Tail tube proteins are shown in salmon and grey with long loops in magenta. The distal tail protein is shown in blue with the N-terminus in green and short loop in orange. (B) Detail of interaction interface. Side chains of residues that form the interaction interface are shown in stick representation and selected interatomic distances are indicated.

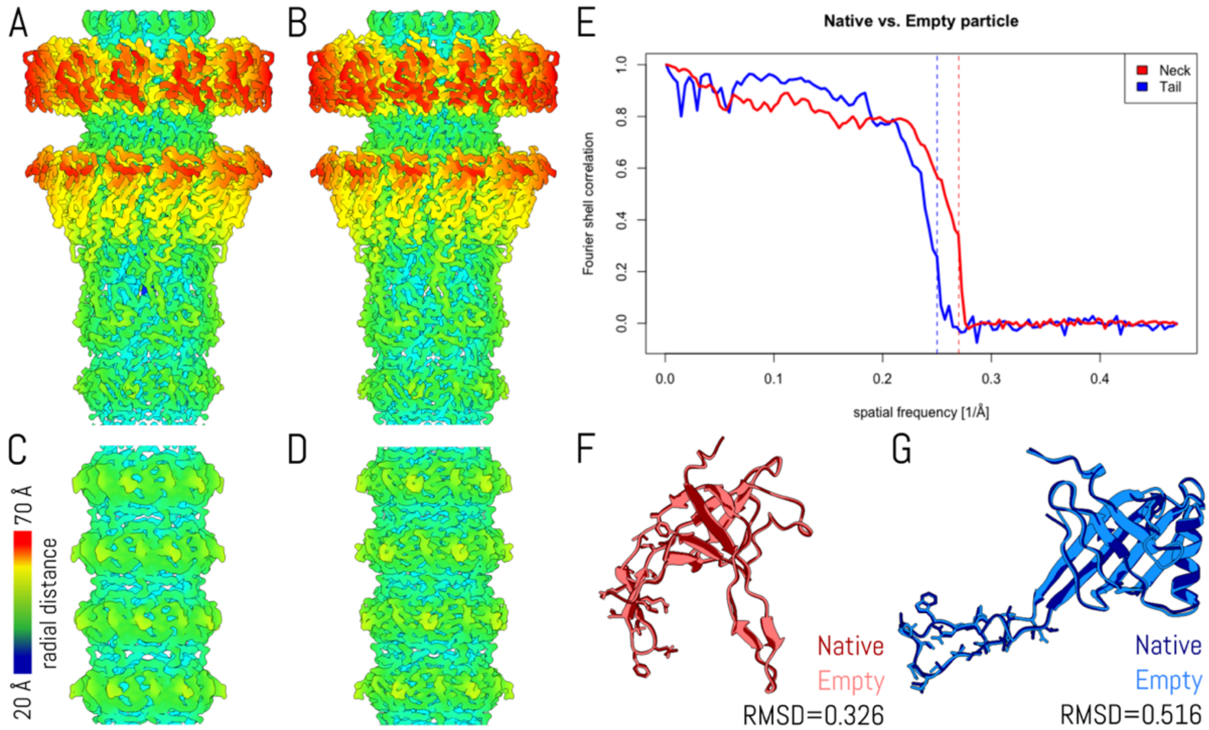


**Supplementary Figure 11. Portal complexes, necks, and tail tubes of native and empty RcGTA particles are nearly identical.** Electron density maps of portal and neck regions (AB) and tail tubes (CD) of native (AC) and empty (BD) RcGTA particles. The maps are rainbow colored based on the distance of the surface from the tail axis, with the scale given on the lower left. (E) Assessment of similarities of necks and tail tubes of native and empty particles by plotting Fourier shell correlation between pairs of the respective structures. Corresponding maps of tail tubes of native and empty particles were low-pass filtered to 4 Å, and maps of neck regions were filtered to 3.7 Å. In both cases the FSC curves indicate high overall similarities above the low-pass limits. (FG) Comparison of structures of stopper (F) and tail tube (G) proteins from native and empty RcGTA particles. RMSD values of corresponding atoms are indicated. Side chains of residues from long loops are displayed in stick representation.

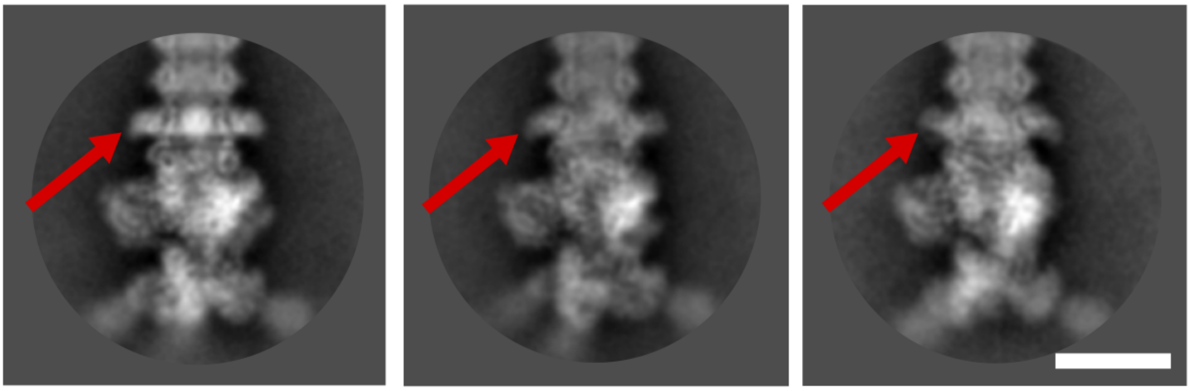

**Supplementary Figure 12. Flexibility of receptor binding domains of distal tail proteins of RcGTA.** Two-dimensional reference-free class averages of RcGTA tails demonstrate the flexibility of insertion domains of distal tail proteins (indicated by red arrows). The reconstructions, from left to right, are based on 1,519; 1,504; and 1,035 particle images, respectively. Scale bar represents 10 nm.

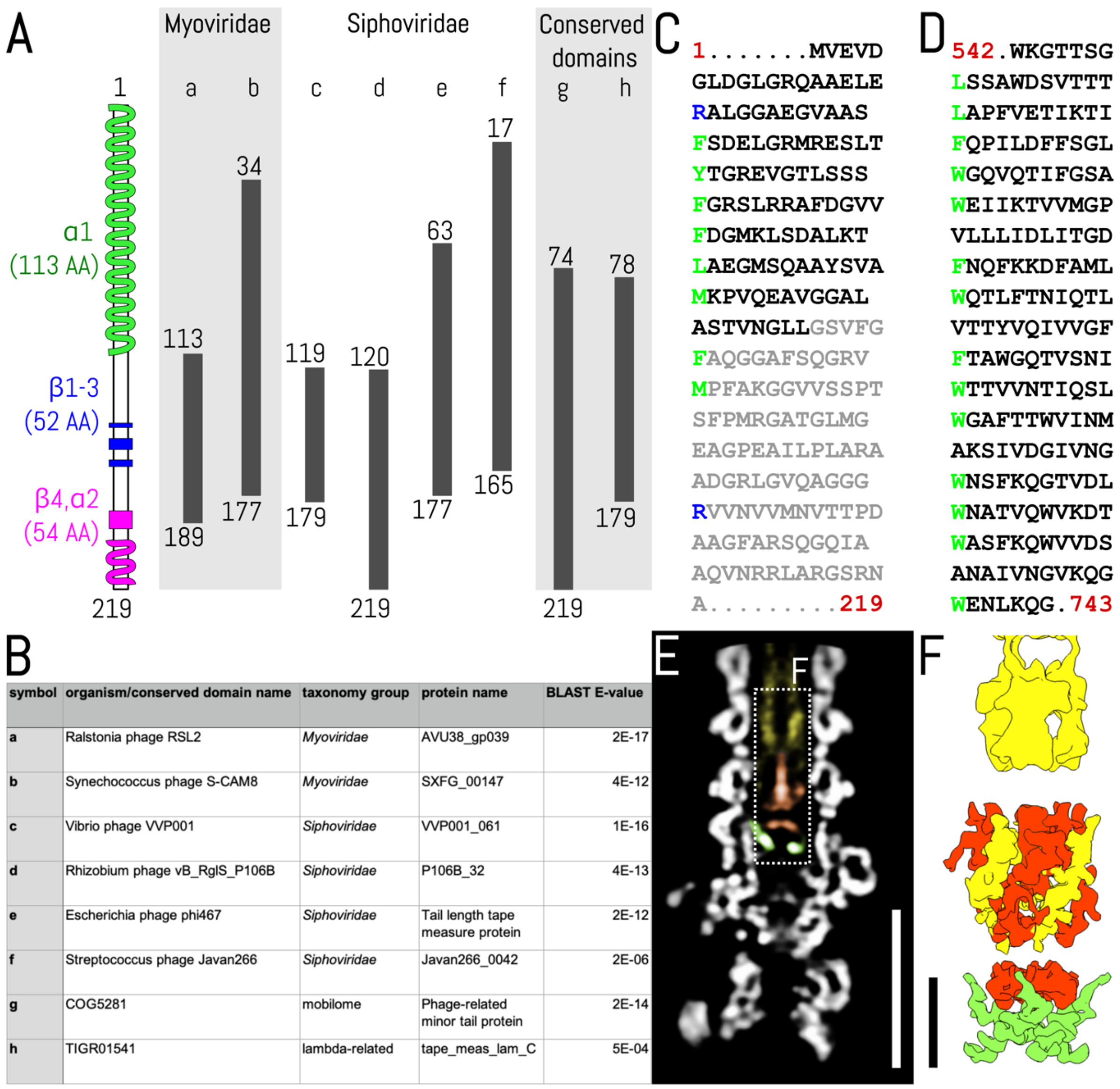


| symbol | organism/conserved domain name | taxonomy group | protein name | BLAST E-value |
|---|---|---|---|---|
| a | Ralstonia phage RSL2 | *Myoviridae* | AVU38_gp039 | 2E-17 |
| b | Synechococcus phage S-CAM8 | *Myoviridae* | SXFG_00147 | 4E-12 |
| c | Vibrio phage VVP001 | *Siphoviridae* | VVP001_061 | 1E-16 |
| d | Rhizobium phage vB_RglS_P106B | *Siphoviridae* | P106B_32 | 4E-13 |
| e | Escherichia phage phi467 | *Siphoviridae* | Tail length tape measure protein | 2E-12 |
| f | Streptococcus phage Javan266 | *Siphoviridae* | Javan266_0042 | 2E-06 |
| g | COG5281 | mobilome | Phage-related minor tail protein | 2E-14 |
| h | TIGR01541 | lambda-related | tape_meas_lam_C | 5E-04 |

**Supplementary Figure 13. Sequence similarity of the tape measure protein of RcGTA (Rcc01694) to those of phages from the families *Siphoviridae* and *Myoviridae*.** (A) Plot of sequence similarities between the RcGTA tape measure protein and those of tailed phages identified using BLASTp(*8, 9*). The names of proteins and phages are listed in panel (B). (B) List of proteins with sequence similarities to the tape measure protein of RcGTA. (CD) Sequence repetitions in coiled-coil region of the tape-measure protein of RcGTA (C) and phage TP901-1 from the family *Siphoviridae* (D) (*10*). The repeats are 12 or 13 residues long, beginning with a large charged (blue) or large hydrophobic (green) side chain. Residues belonging to the predicted α-helical region of the tape measure proteins are shown in black and the remaining residues in grey font. (EF) Cryo-EM density filling tail channel of native RcGTA particles corresponding to: trimer of tape measure proteins (yellow), monomer of peptidase (orange), and three N-termini of megatron proteins (green). (E) Central slice through cryo-EM density of end of RcGTA tail. The reconstruction is based on 26781 particle images. Scale bar represents 10 nm. (F) Surface representation of internal tail density. Scale bar represents 2 nm.

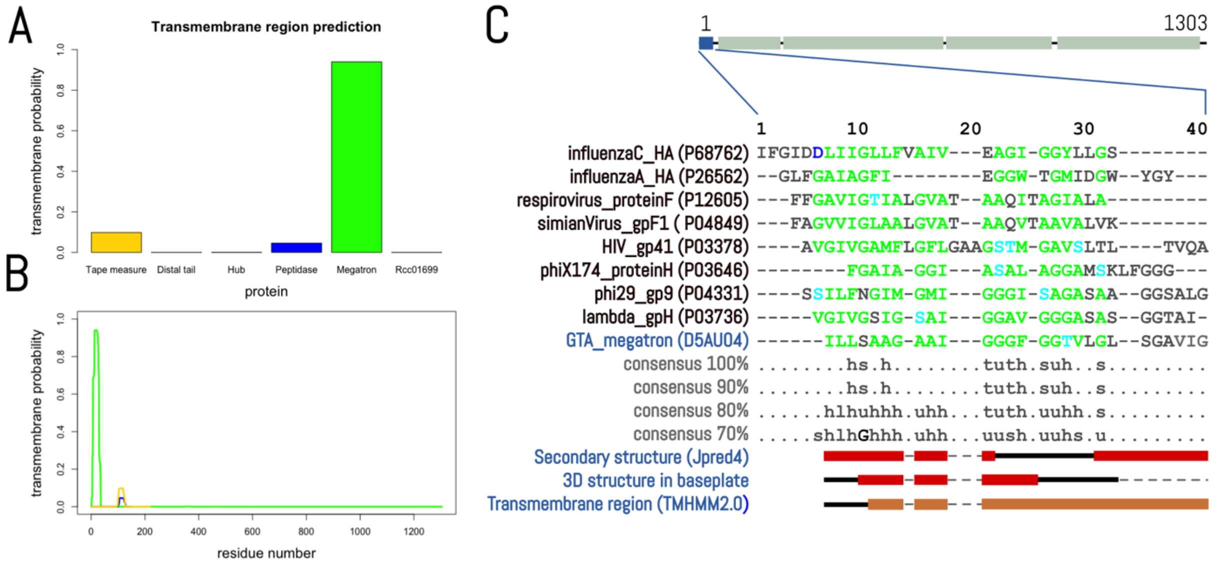


**Supplementary Figure 14. The megatron protein of RcGTA contains a predicted membrane penetration helix.** (A) Probabilities that baseplate proteins of RcGTA contain transmembrane helices as determined by TMHMM2.0 server(*11*). Only the values for the megatron protein are above the threshold. (B) Per-residue plot of probability that residues can form a transmembrane helix. The plot for tape measure protein is shown in yellow, peptidase in blue, and megatron protein in green. (C) Multiple sequence alignment of iris/penetration domain of RcGTA megatron protein with previously identified virus membrane-penetration peptides prepared using the program PROMALS3D(*12, 13*). Conserved amino acids with the predominant property are colored in green, with the minor property in cyan. At the bottom, a representation of parts of the structure forming $\alpha$-helices as determined based on: (1) secondary structure prediction using the program Jpred4(*14*), (2) cryo-EM structure, and (3) prediction of transmembrane helices using the program TMHMM2.0(*11*).

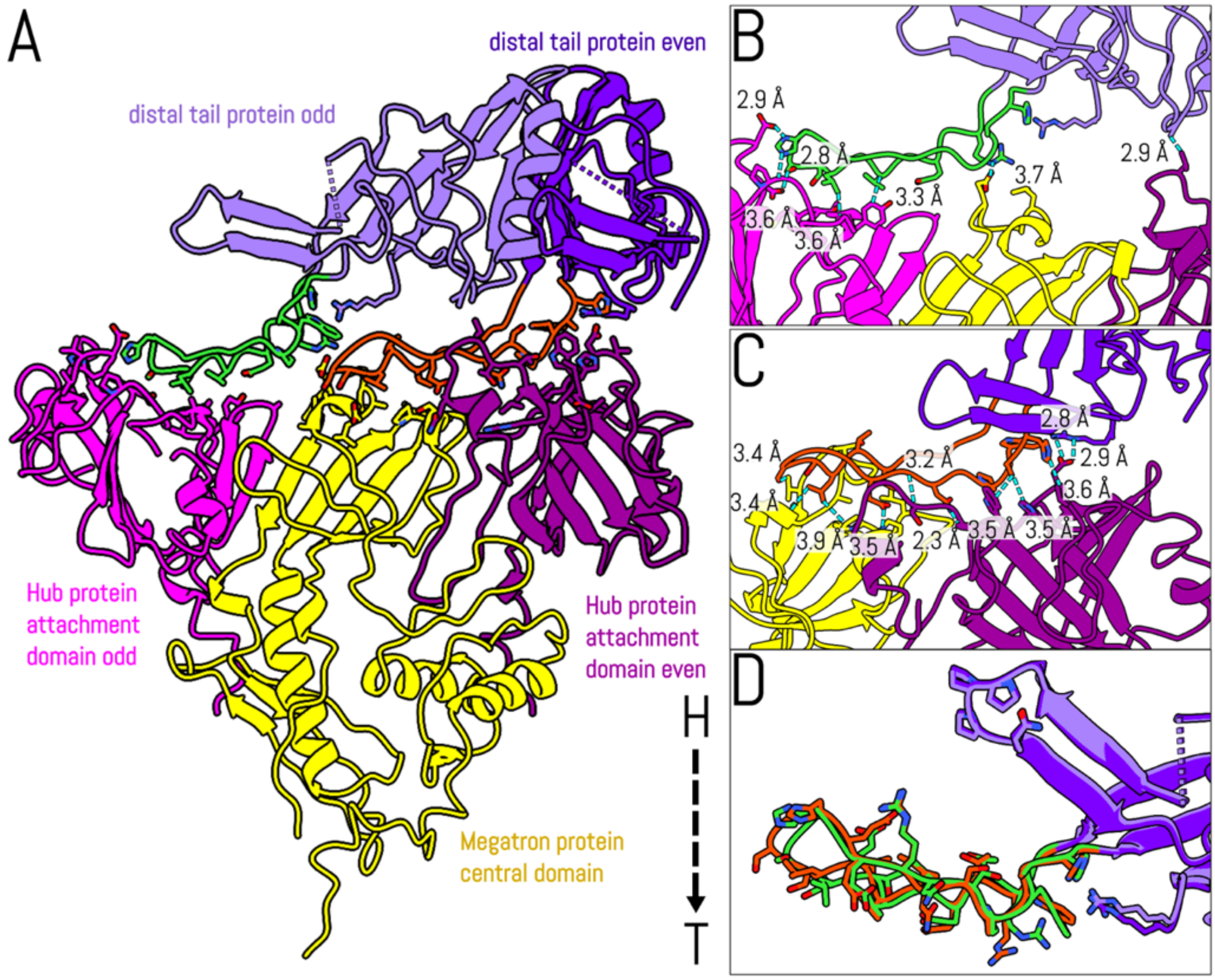


**Supplementary Figure 15. Interface between distal tail proteins and baseplate of RcGTA.** (A) Interaction of two distal tail proteins (distinguished by light and dark blue) with attachment domains of two hub proteins (differentiated by light and dark magenta) and the central domain of the megatron protein (yellow). Long loops of the two distal tail proteins are highlighted in green and orange. The head-to-tail direction is indicated with the black dashed arrow. (BC) Asymmetric interactions of distal tail proteins with baseplate. One of the distal tail proteins interacts predominantly with the hub protein (B), and the other one with both hub and megatron proteins (C). (D) Conformational differences of long loops of two neighboring distal tail proteins.

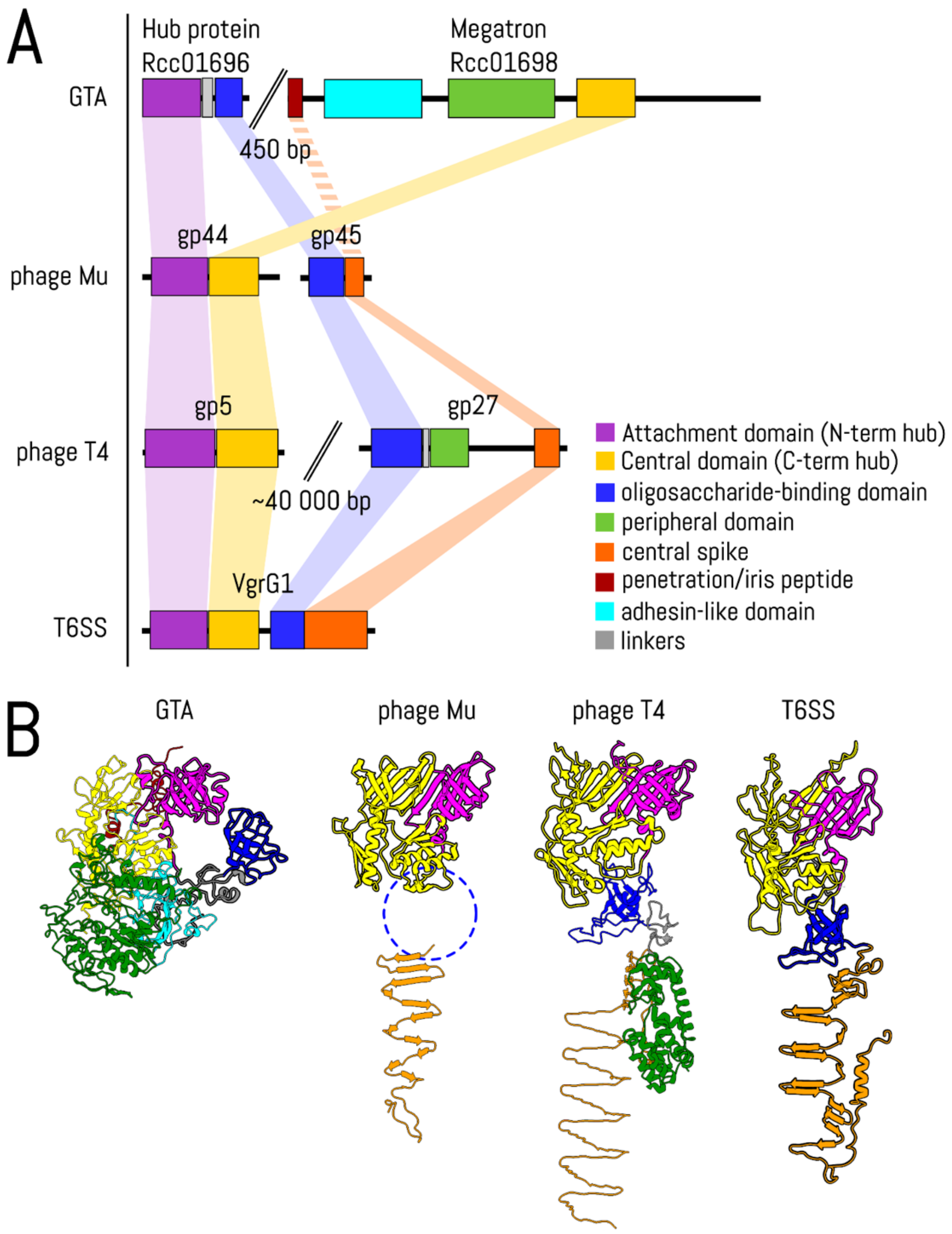


**Supplementary Figure 16. Structural similarities of hub and megatron proteins of RcGTA to those of bacteriophages and a bacterial secretion system.** (A) Distribution of homologous domains in baseplate proteins of RcGTA, phages Mu and T4, and the T6 secretion system of *Pseudomonas aeruginosa*, indicating numerous examples of domain swapping. Individual domains within the proteins used for comparison are annotated based on their presence in experimentally determined structures or identification in sequences using Pfam domain search(*15*). Connections by unbroken color bands highlight the homology of domains. A dashed connection indicates that the penetration domain of the megatron protein of RcGTA, the central spikes of phages Mu and T4, and the T6 secretion system have different structures but serve the same function in forming pores through bacterial outer membranes. (B) Comparison of structures of baseplate proteins of GTA, phages Mu and T4, and the T6 secretion system. Domains are colored according to the convention used in panel (A). The structure of the oligosaccharide-binding domain of phage Mu is not known, however, the sequence prediction indicates that it is part of the baseplate and its putative position is indicated by a dashed blue circle.

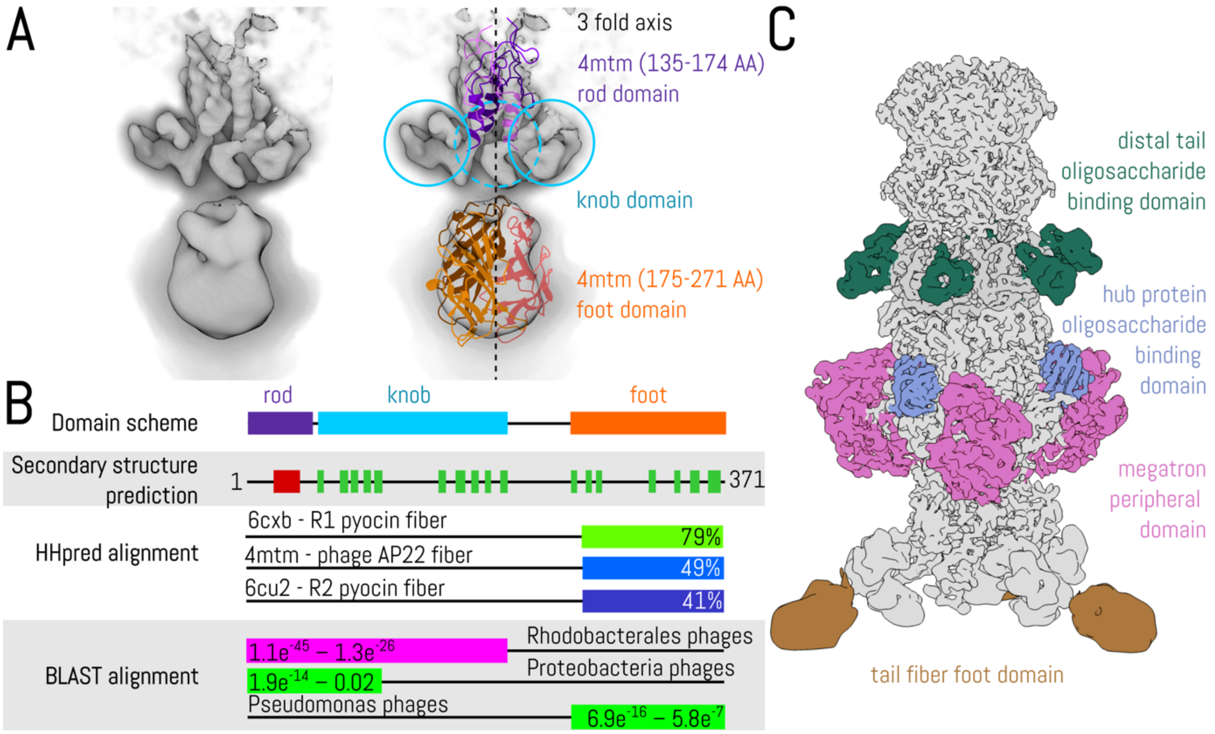


**Supplementary Figure 17. Receptor-binding sites in RcGTA tail.** (A) Map of tail fiber reconstructed to a resolution of 6.8 Å for the region proximal to the baseplate (upper) and 13.9 Å for the distal part (lower). The map of the RcGTA fiber can be interpreted by fitting tail fibers of phage AP22 (PDB 4mtm), however, the RcGTA structure contains extra knob domains (highlighted by cyan circles). (B) Sequence analysis of RcGTA tail fiber protein: domains and secondary structures predicted with the program Jpred4(*14*); secondary structure alignment against structures available from Protein Data Bank identified with HHpred(*16*); and primary sequence alignment against sequences of tailed phages determined using BLASTp(*9*). HHpred probability values and BLASTp E-values are indicated(*9, 16*). (C) Cryo-EM reconstruction of end of RcGTA tail and baseplate with highlighted putative receptor-binding domains: six in the distal tail proteins (green), three in the hub proteins (blue), three in the megatron proteins (magenta), and nine in the tail fibers (each tail fiber is a trimer) (brown).

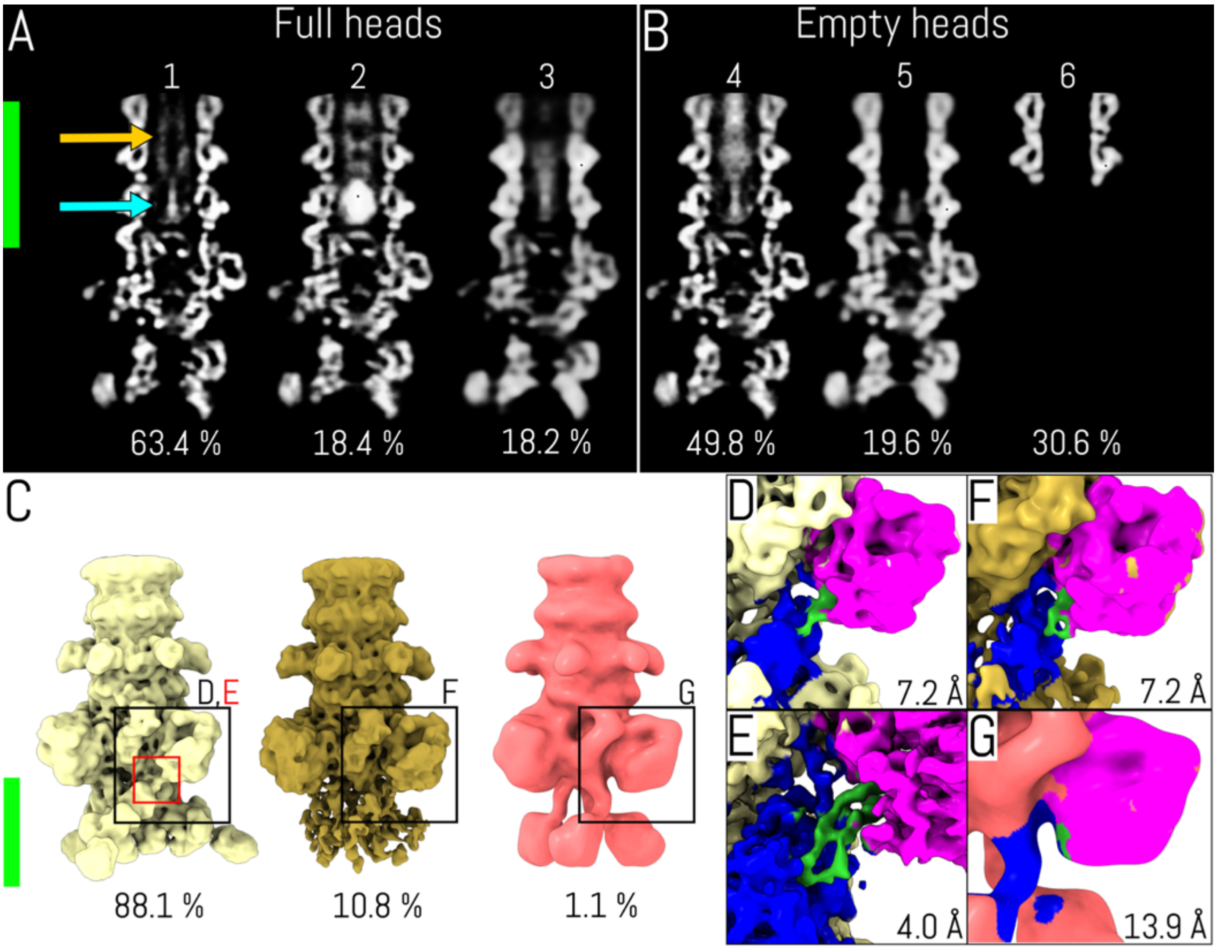


**Supplementary Figure 18. Structural variability of RcGTA baseplates.** (AB) Central sections of three-dimensional reconstructions of RcGTA baseplates of native (A) and empty (B) particles. Images used for the reconstructions were classified based on the region corresponding to the central density of the tail (indicated by a green bar on the left of panel A). Focusing the classification on this region enabled the differentiation of particles based on the presence of tape-measure protein and peptidase, indicated by yellow and cyan arrows respectively. Three biologically relevant classes were obtained for both native and empty particles. (A) 63.4% of the baseplates of native particles contained both tail tube protein and peptidase, 18.4% contained high density in the peptidase region, and 18.2% contained weak and blurred density in the tail tube. (B) 49.8% of baseplates of the empty particles contained peptidase and disordered density in the region of tape-measure proteins, 19.5% lacked tape-measure proteins, and 30.6% lacked baseplates. (C) Three-dimensional reconstructions of baseplates of native RcGTA particles classified according to the volume corresponding to megatron fiber-binding domains and tail fibers (indicated by a green bar on the left of the panel). The contour level is the same for all three maps. 88.1% of particles contained a resolved density of tail fibers, 10.8% of particles contained a limited density of tail fibers, and in 1.1% of particles the tail fibers were shifted away from the core of the baseplate. Squares in panel (C) indicate magnified regions shown in panels D-G. (D-G) Details of peripheral and fiber-binding domains of megatron proteins that are proposed to be responsible for the attachment and positioning of tail fibers. The electron density that stabilizes tail fibers in the native conformation is well resolved in native baseplates (DE), bent in baseplates with flexible fibers (F), and missing in the reconstruction with tail fibers shifted away from the baseplate (G).

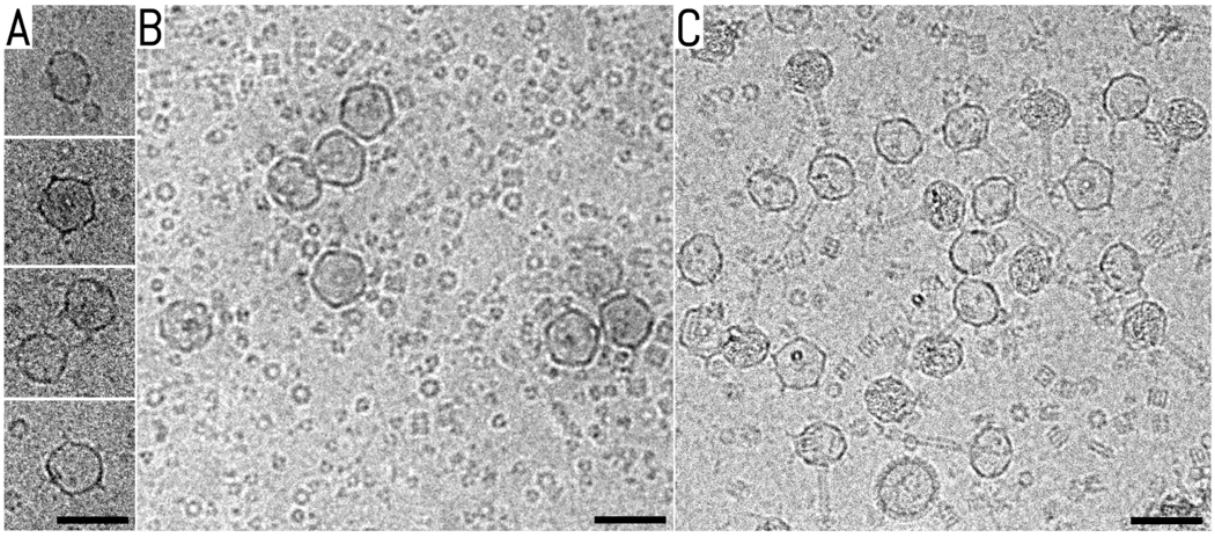


**Supplementary Figure 19. Tail peptidase (g14) is required for assembly of RcGTA tail.** (AB) Knock-out strain SB1003 in peptidase gene (Rcc01697, g14) produced empty heads without tails, suggesting that the peptidase is essential for tail formation. (A) In total, twelve particles produced by *R. capsulatus* strain SB1003wt_KO1697 were observed. (B) In total, 225 particles produced by *R. capsulatus* strain SB1003_overproducer_KO1697 were observed. (C) Particles of wild-type RcGTA. In total, 69,220 particles produced by *R. capsulatus* strain DE442 were observed. Scale bar represents 50 nm.

## Supplementary Tables:

**Supplementary Table 1. Cryo-EM structure quality indicators.**

| Parameters | Native particle | | | | | | | | | Empty particle | | | | | | |
|---|---|---|---|---|---|---|---|---|---|---|---|---|---|---|---|---|
| | capsid c5 | capsid c1 | neck c12 | neck c6 | tail c6 | tail c3 | base c6 | base c3 | base c1 | capsid I4 | capsid c5 | capsid c1 | neck c12 | neck c6 | tail c6 | base c3 |
| **Data collection and processing** | | | | | | | | | | | | | | | | |
| Magnification | 75000 | 75000 | 75000 | 75000 | 75000 | 75000 | 75000 | 75000 | 75000 | 75000 | 75000 | 75000 | 75000 | 75000 | 75000 | 75000 |
| Voltage [kV] | 300 | 300 | 300 | 300 | 300 | 300 | 300 | 300 | 300 | 300 | 300 | 300 | 300 | 300 | 300 | 300 |
| Exposure [e-/Å2] | 42.75 | 42.75 | 42.75 | 42.75 | 42.75 | 42.75 | 42.75 | 42.75 | 42.75 | 42.75 | 42.75 | 42.75 | 42.75 | 42.75 | 42.75 | 42.75 |
| Pixel size | 1.063 | 1.063 | 1.063 | 1.063 | 1.063 | 1.063 | 1.063 | 1.063 | 1.063 | 1.063 | 1.063 | 1.063 | 1.063 | 1.063 | 1.063 | 1.063 |
| Symmetry | C5 | C1 | C12 | C6 | C6 | C3 | C6 | C3 | C1 | I4 | C5 | C1 | C12 | C6 | C6 | C3 |
| Init. No of particles | 53432 | 39403 | 41516 | 41516 | 41806 | 41806 | 42242 | 42242 | 42242 | 1076 | 85271 | 47071 | 49821 | 49821 | 49821 | 56954 |
| Final No. of particles | 39403 | 35966 | 41151 | 41516 | 41806 | 27724 | 37230 | 42242 | 41858 | 898 | 47071 | 28934 | 35512 | 49821 | 49821 | 26978 |
| Initial model | C5 map of empty capsid, 50 Å low-pass | C5 map of full capsid, 50 Å low-pass | C12 map of empty neck, 50 Å low-pass | C12 map of full neck, 40 Å low-pass | C3 map of full tail, 12 Å low-pass | relion initial helix | C3 map of full baseplate, 50 Å low-pass | C3 map of empty baseplate, 50 Å low-pass | C3 map of full baseplate, 50 Å low-pass | de novo | de novo | C1 map of full capsid, 40 Å low-pass | de novo | C12 map of empty neck, 20 Å low-pass | relion initial helix | de novo |
| Map resolution [Å] | 3.56 | 4.25 | 3.32 | 3.58 | 3.89 | 4.54 | 4.14 | 3.99 | 4.56 | 4.03 | 3.42 | 4.12 | 3.36 | 3.47 | 3.77 | 4.49 |
| FSC threshold | 0.143 | 0.143 | 0.143 | 0.143 | 0.143 | 0.143 | 0.143 | 0.143 | 0.143 | 0.143 | 0.143 | 0.143 | 0.143 | 0.143 | 0.143 | 0.143 |
| **Database entry** | | | | | | | | | | | | | | | | |
| EMDB | EMD-10442 | EMD-10568 | EMD-10476 | EMD-10477 | EMD-10478 | EMD-10570 | EMD-10479 | EMD-10490 | EMD-10572 | EMD-10567 | EMD-10565 | EMD-10569 | EMD-10541 | EMD-10542 | EMD-10566 | EMD-10571 |
| PDB | 6TB9 | 6TBA* | 6TE8 | 6TE9 | 6TEA | - | 6TEB | 6TEH | - | 6TSW | 6TSU | XXX | 6TO8 | 6TOA | 6TSV | - |
| **Refinement** | | | | | | | | | | | | | | | | |
| Atoms (except hydrogens) | 70638 | - | 4097 | 11151 | 3888 | - | 4056 | 7545 | - | 6591 | 70607 | - | 4097 | 11316 | 3972 | - |
| Residues | 9465 | | 542 | 1482 | 520 | | 528 | 999 | | 885 | 9461 | | 542 | 1501 | 536 | |
| B-factors | 51.56 | - | 39.79 | 70.99 | 151.64 | - | 128.39 | 69.72 | - | 74.36 | 39.47 | - | 42.29 | 50.99 | 81.90 | - |
| **RMSD** | | | | | | | | | | | | | | | | |
| Bond lenghts [Å] | 0.009 | - | 0.004 | 0.011 | 0.008 | - | 0.007 | 0.009 | - | 0.008 | 0.009 | - | 0.009 | 0.009 | 0.011 | - |
| Bond angles [°] | 1.397 | - | 0.709 | 1.532 | 1.331 | - | 1.329 | 1.366 | - | 1.134 | 1.201 | - | 1.427 | 1.455 | 1.441 | - |
| **Validation** | | | | | | | | | | | | | | | | |
| MolProbity score | 2.01 | - | 2.13 | 2.13 | 2.15 | - | 2.51 | 2.30 | - | 2.15 | 2.16 | - | 1.97 | 2.09 | 2.07 | - |
| ClashScore | 8.10 | - | 12.42 | 11.52 | 10.86 | - | 17.9 | 13.36 | - | 11.23 | 11.75 | - | 8.65 | 9.23 | 0.49 | - |
| C-beta outliers | 0 | - | 0 | 0 | 0 | - | 0 | 0 | - | 0 | 0 | - | 0 | 0 | 0 | - |
| Poor rotamers [%] | 0.63 | - | 0.98 | 0.82 | 0 | - | 1.20 | 0.95 | - | 0.92 | 0.5 | - | 0.98 | 0.54 | 0.00 | - |
| **Ramachandran plot** | | | | | | | | | | | | | | | | |
| outliers [%] | 0.29 | - | 0.38 | 0.07 | 0.00 | - | 0.20 | 0.20 | - | 0.11 | 0.22 | - | 0.19 | 0.14 | 0.00 | - |
| favored [%] | 89.22 | - | 90.79 | 89.99 | 88.10 | - | 83.01 | 84.91 | - | 88.60 | 88.89 | - | 91.54 | 88.03 | 89.93 | - |

**Supplementary Table 2. List of structural proteins of RcGTA.**

| Protein nomenclature 1 | Protein nomenclature 2 | Protein function | Length [AA] | MW [kDa] | Number of copies in AU | MS-identification | Residues modeled | Domain | Sequence | Model building | Homologous structure | Symmetry of map | Map resolution [Å] | DALI search Z-score | DALI search RMSD [Å] | DALI search sequence length [%] |
|---|---|---|---|---|---|---|---|---|---|---|---|---|---|---|---|---|
| Rcc00171 | DUF2793, ribonuclease III | Tail fiber | 371 | 38.9 | 9 | Yes | - | Rod | 1—45 | No | tail fiber of phage AP22 (4mtm_A) | C3(C1) | 6.8 | | - | |
| | | | | | | | | Knob | 46—259 | No | - | | 6.8-13.9 | | - | |
| | | | | | | | | Foot | 260—371 | No | tail fiber of phage AP22 (4mtm_A) | | 13.9 | | - | |
| Rcc01079 | GhsA | Head-spike base | 84 | 9.1 | 55 | Yes | 1 - 84 | Head-spike base | 1—84 | Yes | penton base of sulfolobus archeal virus (3j31_Q) | C5(C1) | 3.4 | 4.3 | 5.4 | 81 |
| Rcc01080 | GhsB | Head-spike fiber | 325 | 32.9 | 11 | Yes | - | Head-spike fiber | 1—325 | RaptorX-CDM | lectin-like protein (5zbt_A) | C5(C1) | - | 5.1 | 3.8 | 38 |
| Rcc01684 | g3 | Portal | 396 | 42.8 | 12 | Yes | 24-393 | Wing | 24—190, 300—362 | Yes | phage G20C portal protein (4zjn_A) | C12 | 3.3 | 18.6 | 4.3 | 79 |
| | | | | | | | | Stem | 191—219, 274—299 | Yes | | | | | | |
| | | | | | | | | Clip | 220—273 | Yes | | | | | | |
| | | | | | | | | Crown | 363—396 | Yes | | | | | | |
| Rcc01687 | g5 | Major capsid protein | 386(298*) | 40,9(31,4*) | 145(175**) | Yes | 89-385 | Prohead peptide | 1— 88 | No | phage HK97 capsid protein (1ohg_A) | C5 | - | | - | |
| | | | | | | | | Mature capsid protein | 89—386 | Yes | phage HK97 capsid protein (1ohg_A) | | 3.3 | 19.4 | 3.2 | 94 |
| Rcc01688 | g6 | Adaptor protein | 197 | 20.9 | 12 | Yes | 1 - 197 | Tube | 9—23, 36—59 141—171 | Yes | phage HK97 neck protein (3jvo_G) | C12 | 3.3 | 10.1 | 2.5 | 45 |
| | | | | | | | | Adaptor loop | 24—35 | Yes | - | C6 | 3.5 | | | |
| | | | | | | | | Attachment | 1—8, 60 — 140 | Yes | - | C12 | 3.3 | | | |
| | | | | | | | | C-terminal hook | 172—197 | Yes | phage HK97 neck protein (3jvo_G) | | | | | |
| Rcc01689 | g7 | Stopper protein | 112 | 12.4 | 6 | Yes | 3 - 112 | Stopper | 1—112 | Yes | phage SPP1 stopper gp16 (2kca_A) | C6 | 3.5 | 5.4 | 3.4 | 75 |
| Rcc01690 | g8 | Tail terminator | 135 | 13.9 | 6 | Yes | 2 - 135 | Tail terminator | 1—135 | Yes | phage SPP1 tail terminator gp17 (2lfp_A) | C6 | 3.5 | 10.2 | 3 | 85 |
| Rcc01691 | g9 | Tail tube | 137 | 14.4 | 30 | Yes | 3 - 136 | Tail tube | 1—137 | Yes | phage lambda tail tube (2k4q_A) | C6 | 3.5 | 6.1 | 6.6 | 77 |
| Rcc01694 | g11 | Tape measure | 219 | 22.2 | 3 | Yes | - | Tape measure | 1—219 | No | phage P22 tail needle gp26 (2poh_A) | C3 | 5.0 | | - | |
| Rcc01695 | g12 | Distal tail protein | 210 | 22.9 | 6 | Yes | 2 - 82, 157-209 | Distal tail | 1—81, 177—210 | Yes | phage T5 distal tail protein (4jmq_C) | C6 | 4.1 | 7.7 | 3.1 | 93 |
| | | | | | | | | Oligosaccharide-binding | 82—176 | No | phage T5 distal tail protein (4jmq_C) | | 5.0 | | - | |
| Rcc01696 | g13 | Hub protein | 296 | 31.7 | 3 | Yes | 4 - 295 | Attachment | 1—142 | Yes | phage T4 gp27 (2z6b_D) | C3 | 4.0 | 6.6 | 11.1 | 89 |
| | | | | | | | | Iron-sulphur cluster | 143—162, 249—263 | Yes | - | | | | - | |
| | | | | | | | | Oligosaccharide-binding | 163—248 | Yes | phage LKA1 tailspike gp49 (4ru4_B) | | | 4.6 | 9.6 | 100 |
| | | | | | | | | C-terminal clip | 264—296 | Yes | - | | | | - | |
| Rcc01697 | g14 | Peptidase | 150 | 16.2 | 1* | No | - | Peptidase | 1—150 | RaptorX-CDM | putative phage lysin (5d74_B) | - | - | 10.3 | 2.9 | 80 |
| Rcc01698 | g15 | Megatron protein | 1304 | 138.4 | 3 | Yes | 2-22, 38-95, 112-229, 745-984 | Iris/penetration | 1—47 | Yes | penetration protein gp9 of phage phi29 (P04331) | C3 | 4.0 | | - | |
| | | | | | | | | Adhesin-like | 48—228 | Yes | immunoglobulin-like domain (5a2f_A) | | 4.0 | 3.2 | 9.7 | 53 |
| | | | | | | | | Peripheral | 229—744 | RaptorX-CDM | endo-beta-1,4-mannase (3civ_A) | | 4.5 | 12.6 | 3.2 | 42 |
| | | | | | | | | Central | 745—984 | Yes | phage T4 gp27 (2z6b_D) | | 4.0 | 8.8 | 2.9 | 80 |
| | | | | | | | | Fiber-binding | 985—1304 | RaptorX-CDM | Interleukin-20 receptor subunit beta (4doh_B) | | 5.0 | 3.5 | 3.5 | 38 |

MW - molecular weight; AU - asymmetric unit; MS - mass spectrometry; RMSD - root-mean-square deviation calculated using DALI server(17).

**Supplementary Table 3. Mass spectrometry identification of proteins forming RcGTA particles.**

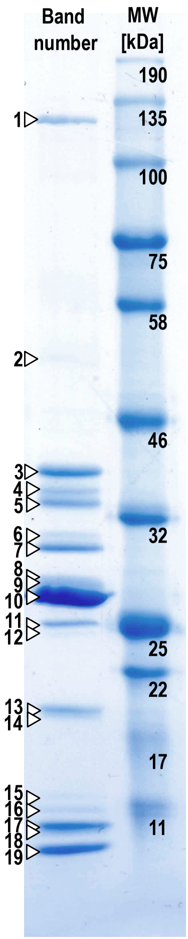


| Band number | Uniprot # | Description | MW [kDa] | calc. pI | SAF | NSAF (%) | Sum(Coverage) | Sum(# Proteins) | Sum(# Peptides) | Sum(# Unique Peptides) |
|---|---|---|---|---|---|---|---|---|---|---|
| 1 | D5AU04 | Megatron (Rcc01698) | 138 | 5.12 | 0.44 | 92.16 | 71% | 1 | 60 | 60 |
| 2 | D5AMD2 | GroEL chaperonin (Rcc02478) | 58 | 5.08 | 0.71 | 71.25 | 75% | 1 | 42 | 42 |
| 3 | D5ATZ0 | Portal (Rcc01684) | 43 | 6.92 | 0.86 | 80.68 | 77% | 1 | 22 | 22 |
| 4 | D5AL80 | Tail fiber (Rcc00171) | 38 | 6.87 | 1.09 | 70.13 | 98% | 1 | 27 | 27 |
| 5 | D5AL80 | Tail fiber (Rcc00171) | 38 | 6.87 | 1.51 | 77.20 | 99% | 1 | 28 | 28 |
| 6 | D5AR34 | Head fibre GTA (Rcc01080) | 33 | 8.63 | 1.21 | 55.77 | 95% | 1 | 22 | 13 |
| | G8GWG3 | Head fibre phage Mu (Rcc00961) | 33 | 6.93 | 0.60 | 27.59 | 36% | 1 | 11 | 2 |
| 7 | D5AR34 | Head fibre GTA (Rcc01080) | 33 | 8.63 | 2.50 | 62.24 | 100% | 1 | 29 | 17 |
| | G8GWG3 | Head fibre phage Mu (Rcc00961) | 33 | 6.93 | 1.20 | 29.95 | 46% | 1 | 15 | 3 |
| 8 | D5AU02 | Hub protein (Rcc01696) | 32 | 5.92 | 0.94 | 60.50 | 85% | 1 | 22 | 22 |
| 9 | D5AU02 | Hub protein (Rcc01696) | 32 | 5.92 | 0.86 | 45.50 | 81% | 1 | 20 | 20 |
| | D5ATZ3 | Major capsid protein (Rcc01687) | 42 | 5.44 | 0.74 | 39.17 | 69% | 1 | 18 | 18 |
| 10 | D5ATZ3 | Major capsid protein (Rcc01687) | 42 | 5.44 | 1.91 | 98.33 | 72% | 1 | 23 | 23 |
| 11 | D5AU01 | Distal tail protein (Rcc01695) | 23 | 6.34 | 1.49 | 77.90 | 97% | 1 | 19 | 19 |
| 12 | D5ATZ3 | Major capsid protein (Rcc01687) | 42 | 5.44 | 0.27 | 37.36 | 64% | 1 | 15 | 15 |
| 13 | D5ATZ4 | Adaptor (Rcc01688) | 21 | 6.58 | 1.76 | 88.74 | 93% | 1 | 14 | 14 |
| 14 | D5ATZ4 | Adaptor (Rcc01688) | 21 | 6.58 | 1.73 | 69.64 | 75% | 1 | 14 | 14 |
| | D5AU01 | Distal tail protein (Rcc01695) | 23 | 6.34 | 0.38 | 15.33 | 76% | 1 | 12 | 12 |
| | D5AU00 | Tape-measure protein (Rcc01694) | 22 | 9.67 | 0.13 | 5.33 | 63% | 1 | 12 | 12 |
| 15 | D5ATZ3 | Major capsid protein (Rcc01687) | 42 | 5.44 | 0.23 | 22.68 | 59% | 1 | 15 | 15 |
| | D5ATZ7 | Tail tube (Rcc01691) | 14 | 4.51 | 0.12 | 12.18 | 46% | 1 | 5 | 5 |
| 16 | D5ATZ7 | Tail tube (Rcc01691) | 14 | 4.51 | 0.71 | 50.63 | 46% | 1 | 6 | 6 |
| 17 | D5ATZ7 | Tail tube (Rcc01691) | 14 | 4.51 | 2.02 | 39.51 | 56% | 1 | 10 | 10 |
| | D5ATZ6 | Tail terminator (Rcc01690) | 14 | 7.25 | 1.82 | 35.61 | 87% | 1 | 8 | 8 |
| | D5ATZ2 | Prohead protease (Rcc01686) | 20 | 8.12 | 0.58 | 11.26 | 63% | 1 | 10 | 10 |
| 18 | D5ATZ5 | Stopper protein (Rcc01689) | 12 | 9.92 | 1.16 | 32.28 | 66% | 1 | 9 | 9 |
| | D5ATZ6 | Tail terminator (Rcc01690) | 14 | 7.25 | 1.16 | 32.13 | 85% | 1 | 6 | 6 |
| 19 | D5AR33 | Head-spike base (Rcc01079) | 9 | 7.43 | 5.25 | 87.64 | 96% | 1 | 9 | 9 |

Proteins from native RcGTA particles were separated on a gradient 10-18% acrylamide gel. The molecular weight of the marker bands is indicated. Nineteen bands were subjected to mass spectrometry analysis. All putative structural proteins, except for peptidase Rcc01697, were confirmed to be virion components. GroEL is an abundant contaminant of the sample. NSAD - normalized spectral abundance factor to the sum of all spectral abundance factors per band; Sum (Coverage) - percentage of protein sequence covered by identified peptides; Sum (#Peptides) - number of peptides assigned to protein group, including peptides that are shared with other protein groups; Sum (#Unique peptides) - number of peptides that are unique to the specific protein group. The presented results are based on mass spectrometry analysis of bands cut from a single acrylamide gel.

**Supplementary Table 4. Homologues of RcGTA head spike protein (Rcc01079) and adaptor protein (Rcc01688).**

| GTA protein | pdb | organism | virus family | protein annotation | Z-score | RMSD [Å] | sequence coverage [%] | Ref. |
|---|---|---|---|---|---|---|---|---|
| head spike base (Rcc01079) | 3j31_Q | *Sulfolobus turreted* icosahedral virus 1 | *Turriviridae* (dsDNA archeal virus) | penton base | 4.3 | 5.4 | 81 | 17 |
| | 5gka_C | *Aichi virus* A846/88 | *Picornaviridae* (+ssRNA eukaryotic virus) | capsid protein | 3.9 | 3.7 | 86 | 18 |
| | 3gzt_B | *Simian rotavirus* A strain RRV | *Reoviridae* (dsRNA eukaryotic virus) | outer capsid glycoprotein | 3.8 | 5.9 | 82 | 19 |
| | 6q5u_R | Enterobacteria virus PR772 | *Tectiviridae* (ssDNA bacterial virus) | penton protein | 3.1 | 3.1 | 69 | 20 |
| | 3j4u_H | *Bordetella virus* BPP-1 | *Podoviridae* (dsDNA bacterial virus) | cementing protein | 2 | 3.2 | 74 | 21 |
| adaptor (Rcc01688) | 3jvo_G | *Escherichia virus* HK97 | *Siphoviridae* (dsDNA bacterial virus) | head-tail connector | 10.1 | 2.5 | 45 | 22 |
| | 5ydn_A | *Escherichia virus* Mu | *Myoviridae* (dsDNA bacterial virus) | neck subunit | 6.6 | 4.2 | 44 | 23 |
| | 5gai_K | *Escherichia virus* P22 | *Podoviridae* (dsDNA bacterial virus) | peptidoglycan hydrolase | 2.9 | 3.8 | 42 | 24 |

Homologs of RcGTA head spike base protein(18-22) and adaptor protein(23-25) were identified using a DALI search(17), with the RcGTA structure as a reference. RMSD - root-mean-square deviation.

**Supplementary Table 5. Domain definition of RcGTA tail proteins.**

| Protein nomenclature | Protein function | Number of residues | Region | Sequence |
|---|---|---|---|---|
| Rcc01689 | Stopper protein | 112 | N-terminus | 1-6 |
| | | | Core β-sheet | 7-12, 32-39, 59-68, 78-92, 100-150 |
| | | | Insertion | 13-31 |
| | | | Long loop | 40-58 |
| | | | Central helix | 69-77 |
| | | | Short loop | 93-99 |
| Rcc01690 | Tail terminator | 135 | N-terminus | 1-26 |
| | | | Insertion | 27-43 |
| | | | Core β-sheet | 44-53, 61-77, 92-116, 120-135 |
| | | | Long loop | 54-60 |
| | | | Central helix | 78-91 |
| | | | Short loop | 117-120 |
| Rcc01691 | Tail tube | 137 | N-terminus | 1-6 |
| | | | Core β-sheet | 7-34, 58-71, 83-112 |
| | | | Long loop | 35-57 |
| | | | Central helix | 72-82 |
| | | | Short loop | 113-117 |
| Rcc01694 | Tape measure | 219 | α-helical core | 1-113 |
| | | | β-helix | 114-165 |
| | | | Lazo | 166-219 |
| Rcc01695 | Distal tail protein | 210 | N-terminus | 1-12 |
| | | | Core β-sheet | 13-21, 44-55, 71-81, 177-193, 197-210 |
| | | | Long loop | 22-43 |
| | | | Central helix | 56-70 |
| | | | Insertion | 82-176 |
| | | | Short loop | 194-196 |

**Supplementary References:**


1. J. Zivanov et al., New tools for automated high-resolution cryo-EM structure determination in RELION-3. Elife 7, (2018).
2. T. D. Goddard et al., UCSF ChimeraX: Meeting modern challenges in visualization and analysis. Protein Sci 27, 14-25 (2018).
3. J. L. Kizziah et al., Cleavage and Structural Transitions during Maturation of Staphylococcus aureus Bacteriophage 80alpha and SaPI1 Capsids. Viruses 9, (2017).
4. A. Fokine et al., Molecular architecture of the prolate head of bacteriophage T4. Proc Natl Acad Sci U S A 101, 6003-6008 (2004).
5. J. Tang et al., Peering down the barrel of a bacteriophage portal: the genome packaging and release valve in p22. Structure 19, 496-502 (2011).
6. D. Hrebik et al., Structure and genome ejection mechanism of Staphylococcus aureus phage P68. Sci Adv 5, eaaw7414 (2019).
7. S. Wang, S. Sun, Z. Li, R. Zhang, J. Xu, Accurate De Novo Prediction of Protein Contact Map by Ultra-Deep Learning Model. PLoS Comput Biol 13, e1005324 (2017).
8. S. F. Altschul, W. Gish, W. Miller, E. W. Myers, D. J. Lipman, Basic Local Alignment Search Tool. J Mol Biol 215, 403-410 (1990).
9. S. F. Altschul et al., Gapped BLAST and PSI-BLAST: a new generation of protein database search programs. Nucleic Acids Res 25, 3389-3402 (1997).
10. J. Mahony et al., Lactococcus lactis phage TP901–1 as a model for Siphoviridae virion assembly. Bacteriophage 6, e1123795 (2016).
11. A. Krogh, B. Larsson, G. von Heijne, E. L. Sonnhammer, Predicting transmembrane protein topology with a hidden Markov model: application to complete genomes. J Mol Biol 305, 567-580 (2001).
12. J. Pei, N. V. Grishin, PROMALS3D: multiple protein sequence alignment enhanced with evolutionary and three-dimensional structural information. Methods Mol Biol 1079, 263-271 (2014).
13. J. Pei, B. H. Kim, N. V. Grishin, PROMALS3D: a tool for multiple protein sequence and structure alignments. Nucleic Acids Res 36, 2295-2300 (2008).
14. A. Drozdetskiy, C. Cole, J. Procter, G. J. Barton, JPred4: a protein secondary structure prediction server. Nucleic Acids Res 43, W389-394 (2015).
15. S. El-Gebali et al., The Pfam protein families database in 2019. Nucleic Acids Res 47, D427-D432 (2019).
16. J. Soding, A. Biegert, A. N. Lupas, The HHpred interactive server for protein homology detection and structure prediction. Nucleic Acids Res 33, W244-248 (2005).
17. L. Holm, P. Rosenstrom, Dali server: conservation mapping in 3D. Nucleic Acids Res 38, W545-549 (2010).
18. D. Veesler et al., Atomic structure of the 75 MDa extremophile Sulfolobus turreted icosahedral virus determined by CryoEM and X-ray crystallography. P Natl Acad Sci USA 110, 5504-5509 (2013).
19. L. Zhu et al., Structure of human Aichi virus and implications for receptor binding. Nature Microbiology 1, (2016).
20. J. Z. Chen et al., Molecular interactions in rotavirus assembly and uncoating seen by high-resolution cryo-EM. P Natl Acad Sci USA 106, 10644-10648 (2009).
21. H. K. N. Reddy, M. Carroni, J. Hajdu, M. Svenda, Electron cryo-microscopy of bacteriophage PR772 reveals the elusive vertex complex and the capsid architecture. eLife 8, (2019).
22. X. Zhang et al., A new topology of the HK97-like fold revealed in Bordetella bacteriophage by cryoEM at 3.5 Å resolution. eLife 2, (2013).
23. L. Cardarelli et al., The crystal structure of bacteriophage HK97 gp6: defining a large family of head-tail connector proteins. J Mol Biol 395, 754-768 (2010).
24. T. Iwasaki et al., Three-dimensional structures of bacteriophage neck subunits are shared in Podoviridae, Siphoviridae and Myoviridae. Genes to Cells 23, 528-536 (2018).

25. A. Cuervo et al., Structures of T7 bacteriophage portal and tail suggest a viral DNA retention and ejection mechanism. Nat Commun 10, 3746 (2019).
