# Supplementary material for: Structure and mechanism of DNA delivery of a gene transfer agent
Source: Nat Commun. 2020 Jun 15;11:3034. doi: 10.1038/s41467-020-16669-9 (PMC7296036; doi:10.1038/s41467-020-16669-9)
Supplement: Supplementary file 3 — Reporting Summary [file 41467_2020_16669_MOESM3_ESM.pdf]

nature research

Corresponding author(s): Pavel Plevka

Last updated by author(s): May 12, 2020

Reporting Summary

Nature Research wishes to improve the reproducibility of the work that we publish. This form provides structure for consistency and transparency in reporting. For further information on Nature Research policies, see our Editorial Policies and the Editorial Policy Checklist.

Statistics

For all statistical analyses, confirm that the following items are present in the figure legend, table legend, main text, or Methods section.

n/a	Confirmed	
☒	☐	The exact sample size (*n*) for each experimental group/condition, given as a discrete number and unit of measurement
☐	☒	A statement on whether measurements were taken from distinct samples or whether the same sample was measured repeatedly
☐	☒	The statistical test(s) used AND whether they are one- or two-sided<br>*Only common tests should be described solely by name; describe more complex techniques in the Methods section.*
☒	☐	A description of all covariates tested
☒	☐	A description of any assumptions or corrections, such as tests of normality and adjustment for multiple comparisons
☒	☐	A full description of the statistical parameters including central tendency (e.g. means) or other basic estimates (e.g. regression coefficient) AND variation (e.g. standard deviation) or associated estimates of uncertainty (e.g. confidence intervals)
☒	☐	For null hypothesis testing, the test statistic (e.g. *F*, *t*, *r*) with confidence intervals, effect sizes, degrees of freedom and *P* value noted<br>*Give P values as exact values whenever suitable.*
☒	☐	For Bayesian analysis, information on the choice of priors and Markov chain Monte Carlo settings
☒	☐	For hierarchical and complex designs, identification of the appropriate level for tests and full reporting of outcomes
☐	☒	Estimates of effect sizes (e.g. Cohen's *d*, Pearson's *r*), indicating how they were calculated

Our web collection on statistics for biologists contains articles on many of the points above.

Software and code

Policy information about availability of computer code

Data collection	Automatic data acquisition was performed using EPU 3.0 software. The focus range was set to -1 to -3 µm. Imaging was done under low-dose conditions with a total dose of 42.75 e-/Å2. Nominal magnification was 75,000 x, resulting in a calibrated pixel size of 1.063 Å. Micrographs were collected using a Falcon 3EC direct electron detector operated in linear mode. Micrographs were acquired as 39 fraction movies. Fractions were aligned and dose-weighted using MotionCorr2(31), and the resulting micrographs were used for further processing. Defocus of the micrographs was estimated using gCTF(32).
Data analysis	Subsets of 1000 full and 1000 empty particles of RcGTA were manually picked using EMAN2 e2boxer.py(33). Particles were extracted with a box size of 512 px, and 2D classification was computed using the program RELION2.1(34). The best-looking class averages were used as templates for autopicking from the whole dataset using the program RELION2.1. The parameters of autopicking were optimized on a subset of 20 micrographs. After autopicking and extraction of the particles, the particles were binned twice using Xmipp(35). Multiple rounds of 2D classification were performed to separate full, empty and icosahedral particles using the program RELION2.1. An initial model with imposed C5 symmetry was generated de novo using the stochastic gradient descent method(36) implemented in RELION2.1. Three-dimensional refinement was performed using 3dautorefine in RELION3(1) followed by three-dimensional classification where the orientational search was omitted (skip_align option), and the particles were classified into four classes. Particles forming the best class were selected for the final reconstruction of binned particles. The reconstruction of the native particle was computed using a mask excluding the density inside the head, whereas the reconstruction of empty particles was performed without masking. The mask was prepared using volume segmentation in UCSF Chimera(37) and the relion_mask_create routine from RELION2.1. After the quality and resolution of the binned images stopped improving, the data were unbinned and the final refinement was performed with local angular searches around the known orientation from the binned refinement. To further improve the resolution, CTF refinement was performed using RELION3. The final refinement was done using the skip_align option in 3dautorefine in RELION3. The final resolution was estimated using the FSC0.143 criterion.<br>Molecular structures were built manually using the software Coot(40). After building the initial models, the map was zoned in UCSF Chimera by applying a 4 Å mask on the main chain and the model was iteratively refined in real space using the program Phenix(41). Models were subsequently refined using NCS constraints and interacting partners in the virion, to prevent inter-molecular atom clashes. During the

iterative refinement process, the molecular geometry was monitored by MolProbity(42) and geometrical outliers were fixed manually using the program Coot. Unique chains of the final model were selected and symmetry expanded in Chimera according to the symmetry of the reconstructed map and deposited in PDB. The models of head spike fiber, peptidase and peripheral and fiber-binding domain of megatron protein were computed using RaptorX contact-dependent modelling(6).

For manuscripts utilizing custom algorithms or software that are central to the research but not yet described in published literature, software must be made available to editors and reviewers. We strongly encourage code deposition in a community repository (e.g. GitHub). See the Nature Research guidelines for submitting code & software for further information.

Data

Policy information about availability of data

All manuscripts must include a data availability statement. This statement should provide the following information, where applicable:

- Accession codes, unique identifiers, or web links for publicly available datasets
- A list of figures that have associated raw data
- A description of any restrictions on data availability

Cryo-EM electron density maps have been deposited in the Electron Microscopy Data Bank, https://www.ebi.ac.uk/pdbe/emdb/ (accession numbers are listed in Supplementary Table 1), and the fitted coordinates have been deposited in the Protein Data Bank, www.pdb.org (PDB ID codes are listed in Supplementary Table 1). The authors declare that all other data supporting the findings of this study are available within the article and its Supplementary Information files or are available from the authors upon request.

Field-specific reporting

Please select the one below that is the best fit for your research. If you are not sure, read the appropriate sections before making your selection.

[x] Life sciences [ ] Behavioural & social sciences [ ] Ecological, evolutionary & environmental sciences

For a reference copy of the document with all sections, see nature.com/documents/nr-reporting-summary-flat.pdf

Life sciences study design

All studies must disclose on these points even when the disclosure is negative.

Sample size	Number of particles used for cryo-EM reconstructions is listed in Supplementary table 1.
Data exclusions	In cryo-EM reconstruction varying fractions of particle images were excluded from the final reconstructions based on previous rounds of 2D and 3D classifications. Numbers of particles retained to the final reconstructions are listed in Supplementary table 1.
Replication	Cryo-EM reconstructions were determined de-novo and resulted in biologically meaningful structures that in many cases enabled building of macromolecular structures. This verifies correctness of the reconstructions and there was no need replicate the reconstructions.
Randomization	Half-sets of particles for gold-standard evaluation of reconstruction quality and resolution were selected randomly.
Blinding	Blinding is not relevant to this study since visual inspection of individual cryo-EM images does not enable and was not used for particle selection.

Reporting for specific materials, systems and methods

We require information from authors about some types of materials, experimental systems and methods used in many studies. Here, indicate whether each material, system or method listed is relevant to your study. If you are not sure if a list item applies to your research, read the appropriate section before selecting a response.

Materials & experimental systems

n/a	Involved in the study	
[x]	[ ]	Antibodies
[x]	[ ]	Eukaryotic cell lines
[x]	[ ]	Palaeontology and archaeology
[x]	[ ]	Animals and other organisms
[x]	[ ]	Human research participants
[x]	[ ]	Clinical data
[x]	[ ]	Dual use research of concern

Methods

n/a	Involved in the study	
[x]	[ ]	ChIP-seq
[x]	[ ]	Flow cytometry
[x]	[ ]	MRI-based neuroimaging
